# A Theoretical Study of Hydrogen Abstraction Reactions in Guanosine and Uridine

**DOI:** 10.3390/ijms24098192

**Published:** 2023-05-03

**Authors:** Kasper F. Schaltz, Stephan P. A. Sauer

**Affiliations:** Department of Chemistry, University of Copenhagen, DK-2100 Copenhagen Ø, Denmark

**Keywords:** DNA damage, OH-radicals, DFT calculations, radiation therapy

## Abstract

All the practically possible hydrogen abstraction reactions for guanosine and uridine have been investigated through quantum chemical calculations of energy barriers and rate constants. This was conducted at the level of density functional theory (DFT) with the ωB97X-D functional and the 6-311++G(2df,2pd) Pople basis set. Transition state theory with the Eckart tunneling correction was used to calculate the rate constants. The results show that the reaction involving the hydrogen labeled C4′ in the ribofuranose part has the largest rate constant for guanosine with the value 6.856 × 10^10^ L s^−1^mol^−1^ and the largest for uridine with the value 3.655 × 10^9^ L s^−1^mol^−1^. Based on the results for these two nucleosides, there is a noticeable similarity between the rate constants in the ribofuranose part of the molecule, even though they are bound to two entirely different nucleobases.

## 1. Introduction

Cancer remains one of the most deadly diseases for humans and therefore attempts are always being made to improve the methods of treatment for cancer. One of the most recent developments is hadron therapy, which is the use of charged particles to destroy cancer-infected cells [1,2]. Hadron therapy uses protons or C^6+^ as the charged particle to damage the molecules in the cells. Statistically when targeting cancer cells with beams of cations it is most likely for the ions to collide with water molecules and generate different radicals. One of these radicals that can be formed is the OH-radical, which can then react with other molecules in the cell such as DNA [3].

Multiple other reactions are also possible and therefore detailed knowledge of the chemical reactions and their reaction mechanisms that lead to DNA damage is of great importance as one strives to improve the selectivity of the radiation treatment in order to reduce the side effects. Currently, it has only been possible to experimentally determine the total rate constants for each DNA nucleobase [4]; therefore, computational efforts are required to further investigate the exact reaction mechanisms.

Previous computational studies (for a more extended review of the literature, see, e.g., [5]) have indicated that the OH-radicals that are formed react with the DNA nucleobases by either abstraction of a hydrogen or by addition to the aromatic rings in the nucleobase [6]. Density functional theory (DFT) calculations showed that addition reactions are more favored than abstraction reactions for uracil [7], while others have shown that the same is the case for guanine [8]. Previously published results from calculations of reactions kinetics in the gas phase by our group also showed that both the addition and abstraction pathways are important for the total rate constant in DNA nucleobases [5,9]. Meanwhile, experimental results have shown that hydrogen abstraction also happens from the ribofuranose part of nucleosides [10,11]. This is one of the reasons for extending our calculations [5,9,12,13] to cover in this work nucleosides instead of just nucleobases. Nucleosides contain the respective nucleobase bound to a ribofuranose molecule and calculations on these molecules will therefore give a better picture concerning what happens in actual DNA, as DNA consists of nucleosides and phosphate groups [14].

In this context, it should be mentioned that this work does not take any solvent effects into account, partly as we want to be able to compare our new results for the nucleotides directly to the results of the previous gas phase study for the nucleobases [5,9]. Solvent effects are of course potentially important in the context of actual DNA in the human body. Solvation has previously been shown to increase the barrier for hydrogen abstraction reactions and make the LUMO orbitals of the nucleobases more compact [8]. Solvent effects were also investigated within our group for reactions with adenine and those results indicated that solvation does have an effect on the rate constants and energetics, but it did not really change the preference of the reaction pathways [12]. However, it was also observed that the standard approach for treating solvation, i.e., the PCM model, did not improve agreement with experimental values and gave results in contrast to explicitly including solvent molecules in the calculation with or without a surrounding PCM environment. Multiple other studies have considered solvent effects on these types of reactions including another one from our group [13], where the solvent effects of water on the reaction between the OH-radical and thymine was studied using again not only the PCM model but also inclusion of one or two explicit water molecules. Similar to our investigation of adenine [12], the different solvent models lead to opposing conclusions. Other studies of solvent effects have been carried out for guanosine and uridine and their nucleobases [8,15,16]. In the present work, however, solvent effects have not been included, because based on our previous studies [12,13] it is not clear, what a sufficiently accurate model for treating solvation would be. Furthermore, experimental studies [10] indicate that for duplex DNA in water the accessible surface area of the hydrogen atoms has an important effect, which none of these solvation models can describe. The focus of this work is instead on extending our previous gas phase studies of the nucleobases by studying with the identical computational protocol the possible hydrogen abstraction reactions that the OH-radicals can initiate with two specific nucleosides in contrast to their respective nucleobases.

Nucleosides consist of two parts, their nucleobase part and then a ribofuranose (RF) part. The nucleosides chosen in this work are guanosine and uridine, which contain the two nucleobases guanine and uracil—a purine and a pyrimidine type nucleobase. The motivation for choosing these nucleosides is that guanine is the largest of the DNA nucleobases while uracil is the smallest and that we cover with them both purine and pyrimidine nucleobases.

**Figure 1:**
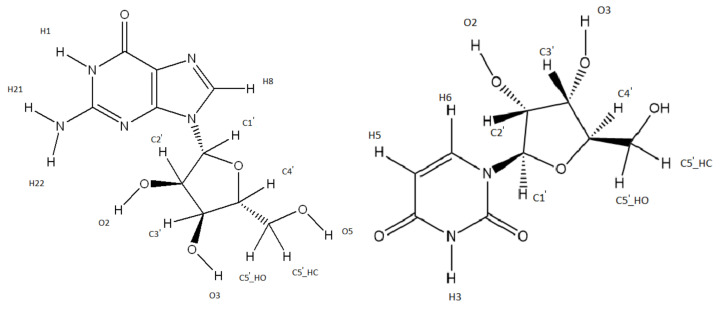
Guanosine and uridine with labels on the hydrogens.

The separation in a nucleobase and ribofuranoce part can also be seen in the chosen labelling of the hydrogens in Figure 1, as two different labelling schemes have been used for the different parts of the nucleosides. In the RF part the hydrogens have been labelled according to which atom they are bound to.

In this project OH-radical hydrogen abstraction reactions were investigated on all but one of the hydrogens in each molecule. The last hydrogens, O5, are replaced by phosphate groups in the actual DNA in a human body and were therefore not investigated, although they were present in the calculations for the other hydrogens. Furthermore, the OH-radical addition reactions to the double bonds in the nucleobases will not be investigated here again.

The aim of the project is to calculate thermodynamic properties of these hydrogen abstraction reactions, calculate the rate constants and then compare them with the results of the previous studies [9,12]. These previous studies investigated all five nucleobases, where the two that are interesting for this study are of course guanine and uracil. In order to accurately compare the results from this study to the previous ones, the computational methods have to be identical. Therefore, the rate constants will be calculated with transition state theory with an Eckart tunneling correction.

The initial intention behind this study was to investigate a couple of different things, one of them being how large the difference between just having the nucleobase in the calculations and also including the ribofuranose molecule is for different properties. This could give a better picture of how accurate all calculations done on just nucleobases are for actual DNA. On top of that, it is interesting, if there is a difference in the properties of ribofuranose, whether it is bound to different nucleobases, which in this case would be guanine and uracil. This might make it possible to predict how ribofuranose will behave when bound to other nucleobases without necessarily running the same amount of calculations.

## 2. Results and Discussion

The standard reaction Gibbs free energies, $\Delta_{r}G$ , of all hydrogen abstractions were calculated as difference between the standard Gibbs free energies of the products and reactions. Correspondingly, the difference in standard Gibbs free energies between the transition state and the reactants, $\Delta G^{‡}$ , was obtained.

**Figure 2:**
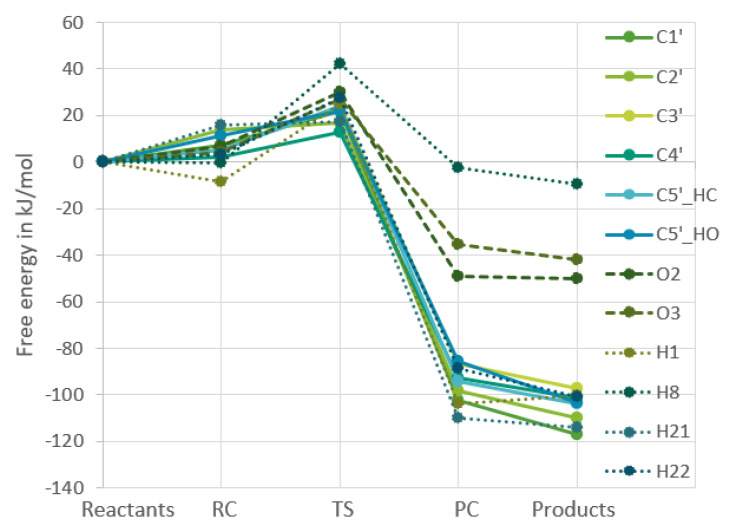
Calculated changes in Gibbs free energy throughout the reaction of guanosine with an OH-radical. All calculations were at the ω B97X-D/6-311G++(2df,2pd) level.

**Figure 3:**
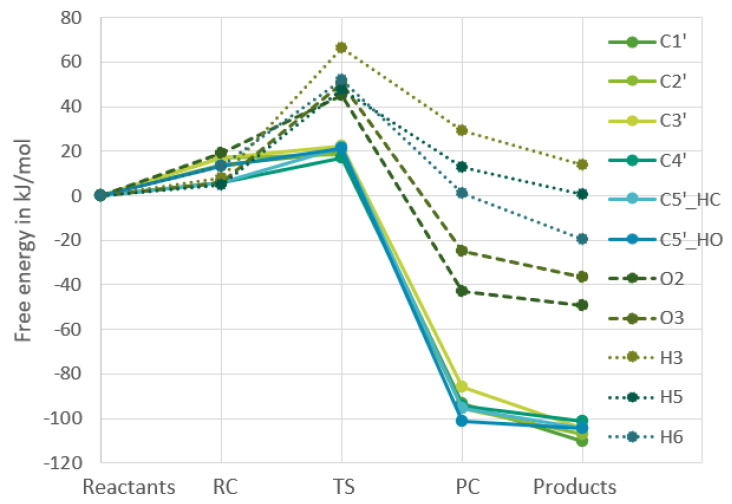
Calculated changes in Gibbs free energy throughout the reaction of uridine with an OH-radical. All calculations were at the ω B97X-D/6-311G++(2df,2pd) level.

Figure 2 and Figure 3 show the change in Gibbs free energy. All of the free energies presented in the plots are relative to the free energy of the reactants, which makes all the reactions for each respective molecule start at the value of 0 kJ/mol, as the starting point of each reaction is the same, namely the free Gibbs energy of an OH-radical and either guanosine or uridine added together.

**Figure 4:**
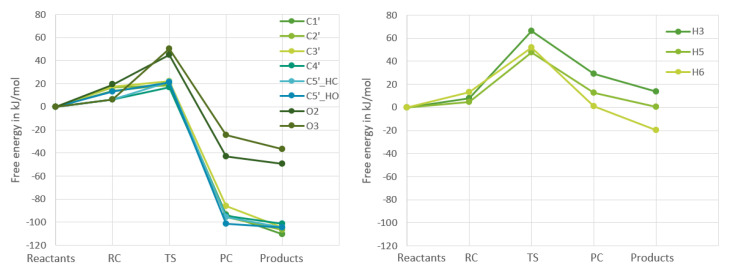
Calculated changes in Gibbs free energy throughout the reaction of the ribofuranose part (**left**) and nucleobase part (**right**) of uridine with an OH-radical. All calculations were at the ω B97X-D/6-311G++(2df,2pd) level.

**Figure 5:**
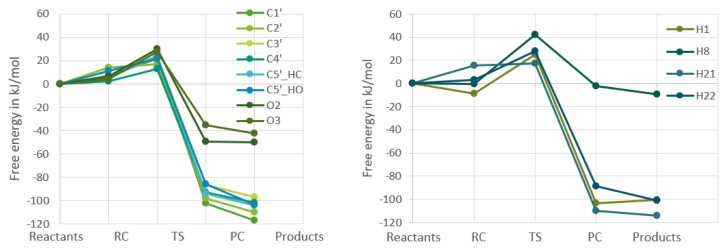
Calculated changes in Gibbs free energy throughout the reaction of the ribofuranose part (**left**) and nucleobase part (**right**) of guanosine with an OH-radical. All calculations were at the ω B97X-D/6-311G++(2df,2pd) level.

The four other points on the plots are the reactant complex, the transition state, the product complex, and the products. From these figures it becomes clear that in both nucleosides the reactions of the hydrogens bound to carbons in the ribofuranose part are very similar in energy, whereas the reactions in the nucleobase part are very different for the two nucleosides, which is to be expected. In Figure 4 and Figure 5, the RF and the nucleobase part are therefore separated into different plots.

Separating the different parts of uridine makes it very clear that the different hydrogen reactions for uridine can be put into three groups regarding the Gibbs free energy. With those three being the ribofuranose hydrogens bound to carbons, ribofuranose hydrogens bound to oxygens and finally the hydrogens bound to the nucleobase. The data indicate that the ribofuranose hydrogens bound to carbons will have the largest reaction rates and be the most thermodynamically stable given that the $\Delta G^{‡}$ values are the smallest and the $\Delta_{r}G$ values the most negative ones for that group.

For guanosine, the two groups that have to do with RF follow the same trend as for uridine. As for the nucleobase part, it is a bit more complicated as one of the nucleobase hydrogens behaves completely differently from the other three as discussed previously [9]. The former three are very similar to the hydrogens bound to the RF part of guanosine, while the H8 reaction is similar to the nucleobase reactions in uridine. However, one would expect from Figure 4 and Figure 5 that the hydrogen abstraction reactions for guanosine will have more similar rate constants than the uridine reactions.

### 2.1. Gibbs Free Energies: $\Delta G^{‡}$ and $\Delta_{r}G$

Looking at the exact values of the Gibbs free energies presented in Table 1, one can attempt to predict the relative sizes of the rate constants by looking at the relative $\Delta G^{‡}$ values. For guanosine, these values are very similar for all reactions except for the H8 hydrogen. The H8 reaction has a higher $\Delta G^{‡}$ which should result in the rate constant being smaller than for the rest of the guanosine reactions. As for uridine, the hydrogens bound to carbons in the RF part have similar values as for the guanosine reactions. The other two groups, which were recognized from the plots, both have higher values for $\Delta G^{‡}$ . The hydrogens bound to oxygens in the RF part and the H5 and H6 hydrogens have around the same values. This should mean that these reactions will have roughly the same rate constant, which should be significantly lower than for the hydrogens bound to carbons in the RF part.

The value of $\Delta_{r}G$ indicates how thermodynamically stable the product is compared to the reactants. Recalling that the product is a radical and a water molecule, one would expect that the radical would no matter the value of $\Delta_{r}G$ be very unstable and quickly react with something different in the cell. Nevertheless, we can compare to the results of a recent ω B97XD/6-31++G** calculation using the PCM model for solvation [17]. The trend in their ΔG for the radicals should be comparable to the trend in our reaction Gibbs free energies $\Delta_{r}G$ . They find a decreasing stability in the radicals C4’ > C1’ > C5’ > C3’ > C2’, while we find C1’ > C2’ > C5’> C4’ > C3’ for guanosine. However, the computational protocols employed in the two calculations differ not only because they used a PCM environment while our calculations are for the gas phase, but more important our basis set is significantly larger, being both a triple zeta basis set and including also second polarization functions on all atoms.

### 2.2. Eckart Correction

Before comparing the rate constants, the relative size of the Eckart tunneling corrections in Table 2 will be discussed. The Eckart tunneling correction includes contributions from the forward and the reverse energy barriers, calculated from the zero-point vibrationally corrected energies. The Eckart corrections were calculated via an in-house unpublished program, which calculates the integral numerically.

Looking at the values of the Eckart corrections, a similar pattern as with the thermodynamic data can be observed. In both molecules, all of the hydrogens bound to carbons in the RF part have Eckart correction values of around 1. As for the other reactions, they mostly have values that are significantly larger than 1, which in some cases makes sense when looking at just the forward and reverse energy barriers. This can explain the higher values of the Eckart correction because having a higher forward energy barrier allows for more possibilities for tunneling, while having a lower reverse energy barrier allows for fewer possibilities for the “reverse” tunneling. However, one should keep in mind that this correction only accounts for the tunneling effects, meaning that a larger tunneling correction could be associated with an otherwise less favorable reaction.

In some reactions, just looking at the forward and reverse barriers is not enough to explain the relative value of the Eckart corrections compared to some of the other reactions, such as, e.g., for the O2 and O3 reactions in guanosine, which have values of 15.05 and 14.26, respectively. They have basically the same forward energy barrier but there is a significant difference in the reverse energy barrier. This means that one cannot just look at the energy barriers and conclude the value of the Eckart correction from that alone, as it also depends on other properties such as the value of the zero-point vibrationally corrected energies, which are very similar in this case.

### 2.3. Rate Constants

In Table 3, we finally present the rate constants calculated for the abstraction of the different hydrogen atoms in guanosine and uridine. The results show that the reaction at C4’, i.e., in the RF part of the nucleoside, has the largest rate constant for both molecules at 6.856 × 10^10^ L s^−1^mol^−1^ and 3.655 × 10^9^ L s^−1^mol^−1^ for guanosine and uridine, respectively, followed by the abstraction of a hydrogen from C2’—again in the RF part. When looking at the other calculated properties, one observes that these reactions also have the lowest ΔG^‡^ values for both molecules. This inverse relationship between the rate constant and ΔG^‡^ can be noticed throughout all the reactions, showing that the energy term in the TST equation for the rate constant, Equation (1), is the dominant term.

From Figure 4, one would expect that the reactions of the hydrogens bound to carbons in the RF part of the molecules would have rate constants that are larger than the hydrogens bound to the oxygens in the RF and the nucleobase part of uridine. From Figure 5, one would instead expect there to be more parity between the different hydrogen abstraction reactions’ rate constants with the exception of the H8 reaction. Both of these expectations turn out to be true when looking at the data in Table 3.

These data imply that the abstraction reactions of two of the hydrogens in the RF part of guanosine and of all the hydrogens bound to carbon in the RF part of uridine are faster and thus more important than the hydrogen abstraction reactions in the corresponding nucleobases. Compared to the fastest OH-radical addition reactions in guanine and uracil with rate constants $1.867\times 10^{10}\;L\;s^{-1}{\mathrm{mol}}^{-1}$ for guanine and $2.939\times 10^{9}\;L\;s^{-1}{\mathrm{mol}}^{-1}$ for uracil [9], these hydrogen abstraction reactions in the RF part of the nucleosides are also clearly competitive.

Another thing to note when comparing the rate constants, is the difference in size of the rate constants belonging to the hydrogen abstraction reactions in the nucleobase parts of the two molecules. For uridine, the rate constants belonging to the hydrogens in the uracil part are negligible compared to the reactions in the RF part. For guanosine, on the other hand, those rate constants are more significant with three of them, for H1, H21 and H22, being just one order of magnitude smaller than the largest rate constant for the whole nucleoside.

We can also compare, in Table 4 and Table 5, our rate constants for the hydrogen abstraction reactions in the nucleobase part of the nucleosides to the previous results for the hydrogen abstraction reactions in the nucleobases themselves [9]. In general, the changes in the rate constants due to the RF part are relatively small. With the exception of the abstraction of H8 in guanosine/guanine, they differ by less than one order of magnitude. For uridine/uracil, the rate constants are larger in the isolated uracil molecule compared to the rate constants for the same hydrogens. Meanwhile, for guanosine/guanine, the opposite is true for all but the H8 hydrogen abstraction reaction.

Based on the result for these to protypical purine and pyrimidine nucleosides, we predict that the rate constants for the hydrogen abstraction reactions in the other three nucleobases, adenine, cytosine and thymine, will also be rather independent of whether or not the nucleobase is a standalone molecule or if it is bound to an RF molecule. The rate constants of nucleobase hydrogen abstraction in the rest of the nucleosides is thus expected to be within an order of magnitude from the results of the previous studies on the nucleobases [9].

We can also compare our results in Table 3 with experimental results obtained for duplex DNA in water [10]. They observed an order of reactivity H5’ > H4’ > H3’ ≈ H2’ ≈ H1’ and could show that it is directly correlated to the solvent-accessible surface area of the hydrogens—an effect which is not included in our calculations. Our results are, in contrast, for the intrinsic reaction kinetics, which consequently can not easily be extracted from experimental data.

Although no research was carried out on the OH-radical addition reactions in the nucleoase part of the nucleosides, and therefore it is unknown whether the rate constants will be higher or lower for those reactions in guanosine and uridine than in guanine and uracil, those rate constants are not expected to be significantly different from the largest one of the hydrogen abstraction reactions. Which means that it is very likely that these OH-radical addition reactions will compete with the C4’ hydrogen abstraction reaction in both guanosine and uridine.

## 3. Materials and Methods

### 3.1. Eckart Tunneling Correction

In this work transition state theory (TST) [18] along with the Eckart tunneling correction was used to calculate the rate constants,

(1) $$k=\kappa \;\sigma \;\frac{k_{b}T}{h}\frac{Q_{TS}}{Q_{NS}Q_{OH}}exp\left(\frac{-\Delta E^{‡}}{k_{b}T}\right)$$

where the TST equation was expressed in terms of partition functions, where $Q_{NS}$ is the partition function of the nucleoside, σ the symmetry factor (which is 2 for non-planar transition state complexes), $\Delta E^{‡}$ is the difference in energy between the transition state complex and the reactants, and κ is the Eckart tunneling correction. In cases where no tunneling correction was included, the value of κ is 1.

The Eckart tunneling correction is temperature-dependent and accounts only for contributions from tunneling,

(2) $$\kappa =\frac{1}{k_{B}T}exp(\frac{-\Delta E_{fwd}}{k_{B}T})\int_{0}^{\infty }P_{QMT}\left(E\right)*exp(\frac{-E}{k_{B}T})dE$$

where $P_{QMT}$ is the probability of quantum mechanical tunneling through an Eckart potential barrier, *E* is the energy and $\Delta E_{fwd}$ is the forward energy barrier. All of these energies are zero-point vibrationally corrected. The equation for this probability was derived by Eckart [19] and is here employed as given in this article,

(3) $$P_{QMT}=1-\frac{cosh(2\pi (\alpha -\beta ))+cosh(2\pi \delta )}{cosh(2\pi (\alpha +\beta ))+cosh(2\pi \delta )}$$

with $\alpha =\frac{1}{2}\sqrt{\frac{E}{C}}$ , $\beta =\sqrt{\frac{E-A}{C}}$ , $\delta =\frac{1}{2}\sqrt{\frac{B-C}{C}}$ , $C=\frac{h^{2}}{8ml^{2}}$ . A and B are related to the forward $\Delta E_{fwd}$ and reverse energy barriers $\Delta E_{rev}$ :

(4) $$A=\Delta E_{fwd}-\Delta E_{rev}$$

(5) $$B=(\sqrt{\Delta E_{fwd}}+\sqrt{\Delta E_{rev}}{)}^{2}$$

It should be noted that the Eckart tunneling correction only accounts for tunneling, which will give some counter-intuitive scaling with regards to, e.g., temperature. Usually when increasing the temperature, one expects that the rate of the reaction goes up, which is also the case here, but when looking at just the Eckart correction, an increase in temperature will lower the correction. This is due to a higher amount of molecules now being in a higher excited state and having therefore the required energy to surpass the energy barrier, without having to resort to tunneling through it. As a consequence, fewer molecules will tunnel through the barrier. However, this is not a relevant observation for this work as the temperature was kept the same for all of the reactions. Furthermore, one can argue that the Eckart correction as employed in the present and our previous work [5,9,12,13] gives too narrow barriers and therefore overestimates the tunneling correction for temperatures below 500 K and that the small curvature correction [20] should rather be employed. However, for the sake of comparability with the our previous studies, we continued to use the Eckart correction in the present study.

### 3.2. Computational Details

Given the size of the molecules and the amount of calculations required for each hydrogen abstraction reaction, density functional theory (DFT) was chosen as the method for the calculations at the ω B97X-D/6-311G++(2df,2pd) [21,22] level. DFT allows for a decent accuracy while keeping the computational costs fairly low compared to other computational methods. The most important point about DFT is choosing an exchange-correlation functional and in our case the functional chosen is a long-range corrected hybrid functional [23,24,25] that includes dispersion terms [26]. In previous studies, this combination was shown to be the most reasonable compromise between accuracy and computational time [5], for this group of molecules.

All calculations were conducted with the Gaussian16 program [27]. With this program, multiple different types of calculations were carried out. First standard geometry optimizations to a minimum were performed, followed by frequency calculations. Then, attempting to find the transition state for all of the different possible hydrogen abstraction reactions, different optimization methods were used with varying success. The first one was the Berny algorithm [28], but that method proved to be unreliable in this type of calculation and was therefore quickly discarded as a possible method. The next attempt was to optimize using the QST2 method [29,30], which is a method that requires the starting geometry and the final geometry as input and then attempts to find the transition-state structure in between. This method proved to be very successful for this project, as it found transition states for 10 out of 12 of the possible hydrogen abstraction reactions for guanosine and all of them for uridine. For the last two reactions, a very similar method, QST3 [29,30], was successful, where the only difference is that it requires a third input, which is a guess for a possible transition state structure. When the transition states had been found, they were followed by Intrinsic Reaction Coordinate (IRC) calculations to evaluate geometries of the reactant complex and product complex of the reactions corresponding to those transition states. Plots of these IRC calculations are given in the Supplementary Materials. Once the IRC calculations finished, the resulting geometries were then optimized to a minimum, and were then chosen as the reactant and product complexes for the calculation of the tunneling corrections. In the calculation of all Gibbs free energies, the harmonic oscillator approximation was employed for all vibrational modes. Some of the vibrational modes of the transition states might not be harmonic and would better be treated by including anharmonic corrections. Such a treatment was, however, beyond the scope of this study and has not been included here.

## 4. Conclusions

We have systematically investigated all the hydrogen abstraction reactions in the two nucleosides, guanosine and uridine, with DFT calculations at the ωB97X-D/6-311G++(2df,2pd) level. The investigation has shown that, in general, the most favorable hydrogen abstraction reactions occur on the carbon-bound hydrogens in the ribofuranose part of the nucleosides and not in the nucleobase part. The fastest hydrogen abstraction reaction is the one that occurs on the C4’ hydrogen, with a rate constant of 6.856 × 10^10^ L s^−1^mol^−1^ for guanosine and 3.655 × 10^9^ L s^−1^mol^−1^ for uridine. These two reactions also have the lowest ΔG^‡^, indicating that the rate constants are dominated by the exponential energy term.

Comparisons made between the results from the nucleobases alone [9], and from the results gathered in this work on the nucleobases in the nucleoside environment, show that the rate constants for those hydrogen abstraction reactions can be mostly considered as independent of whether or not the nucleobase is a standalone molecule.

Comparing the rate constants of the hydrogen abstractions in the nucleosides with the rate constants for the OH-radical addition reactions to the nucleobases shows that they are of the same order of magnitude and thus both important, supporting thus the previous findings for the nucleobases alone [9].

Further calculations including the phosphate groups, that are bound to guanosine and uridine instead of the O5 hydrogen, could be attempted in future work, although that might require sacrificing some accuracy in the computational method in order to reduce the computational cost. Another possibility for future work would be to investigate how much the rate constants of the OH-radical addition reactions in guanosine and uridine are actually affected by the RF part.

## Figures and Tables

**Table 1:** $\Delta G^{‡}$ and $\Delta_{r}G$ values calculated at the ω B97X-D/6-311G++(2df,2pd) level in kJ/mol.

Guanosine Hydrogen	$\Delta G^{‡}$	$\Delta_{r}G$	Uridine Hydrogen	$\Delta G^{‡}$	$\Delta_{r}G$
C1’	21.86	−117.1	C1’	19.43	−110.5
C2’	16.76	−110.0	C2’	18.67	−107.0
C3’	24.03	−97.16	C3’	22.41	−104.6
C4’	12.82	−101.3	C4’	16.83	−101.3
C5’_HC	23.89	−103.8	C5’_HC	21.54	−104.6
C5’_HO	21.80	−103.4	C5’_HO	21.08	−104.6
O2	29.94	−49.98	O2	44.81	−49.29
O3	27.25	−42.17	O3	50.31	−36.51
H1	25.14	−100.52	H3	66.18	13.92
H8	42.03	−9.39	H5	47.83	0.5437
H21	17.59	−114.00	H6	51.75	−19.85
H22	27.76	−100.99	N/A	N/A	N/A

**Table 2:** Forward energy barriers, reverse energy barriers and Eckart corrections calculated at the ω B97X-D/6-311G++(2df,2pd) level.

Guanosine Hydrogen	Forward Energy Barrier (kJ/mol)	Reverse Energy Barrier (kJ/mol)	Eckart Correction	Uridine Hydrogen	Forward Energy Barrier (kJ/mol)	Reverse Energy Barrier (kJ/mol)	Eckart Correction
C1’	11.229	116.530	1.02	C1’	0.053	107.238	0.72
C2’	−1.764	112.584	1.00	C2’	−1.806	111.547	1.00
C3’	11.959	107.168	1.02	C3’	−0.412	97.755	1.00
C4’	4.001	97.768	1.14	C4’	6.091	99.669	1.31
C5’_HC	14.467	110.940	1.03	C5’_HC	13.889	111.807	1.05
C5’_HO	3.148	97.952	1.14	C5’_HO	2.095	118.861	1.06
O2	20.647	76.279	15.05	O2	21.618	87.784	12.55
O3	19.959	54.371	14.26	O3	40.422	71.487	55.17
H1	29.818	123.136	12.06	H3	57.913	35.505	24.23
H8	43.843	39.351	22.83	H5	40.700	27.909	18.19
H21	0.509	127.731	0.82	H6	32.186	44.547	27.44
H22	23.853	115.021	19.27	N/A	N/A	N/A	N/A

**Table 3:** Rate constants for the different hydrogens in guanosine and uridine (L s^−1^mol^−1^) calculated at the ωB97X-D/6-311G++(2df,2pd) level.

Hydrogen	Guanosine	Hydrogen	Uridine
C1′	1.603 × 10^9^	C1′	7.037 × 10^8^
C2′	1.225 × 10^10^	C2′	1.326 × 10^9^
C3′	6.667 × 10^8^	C3′	2.942 × 10^8^
C4′	6.856 × 10^10^	C4′	3.655 × 10^9^
C5′_HC	7.123 × 10^8^	C5′_HC	4.380 × 10^8^
C5′_HO	1.830 × 10^9^	C5′_HO	5.316 × 10^8^
O2	9.078 × 10^8^	O2	4.386 × 10^5^
O3	2.543 × 10^9^	O3	2.096 × 10^5^
H1	5.034 × 10^9^	H3	1.527 × 10^2^
H8	1.048 × 10^7^	H5	1.878 × 10^5^
H21	7.202 × 10^9^	H6	5.836 × 10^4^
H22	2.795 × 10^9^	N/A	N/A

**Table 4:** Comparison of rate constants in guanosine and guanine (L s^−1^mol^−1^).

Hydrogen	Guanosine	Guanine
H1	5.034 × 10^9^	6.925 × 10^8^
H8	1.048 × 10^7^	8.732 × 10^5^
H21	7.202 × 10^9^	1.036 × 10^10^
H22	2.795 × 10^9^	7.347 × 10^8^

**Table 5:** Comparison of rate constants in uridine and uracil (L s^−1^mol^−1^).

Hydrogen	Uridine	Uracil
H3	1.527 × 10^2^	2.430 × 10^2^
H5	1.878 × 10^5^	2.987 × 10^5^
H6	5.836 × 10^4^	9.281 × 10^4^

## Data Availability

The data that support the findings of this study are available within the article and the Supporting Information. Further data are available from the corresponding author upon reasonable request.

## Supplementary Materials

The following supporting information can be downloaded at: https://www.mdpi.com/article/10.3390/ijms24098192/s1.

## Abbreviations

The following abbreviations are used in this manuscript:

DFT	Density functional theory
RF	Ribofuranose
TST	Transition state theory
