# Correction: Schaltz et al. A Theoretical Study of Hydrogen Abstraction Reactions in Guanosine and Uridine. *Int. J. Mol. Sci.* 2023, *24*, 8192

**DOI:** 10.3390/ijms252212272

**Published:** 2024-11-15

**Authors:** Kasper F. Schaltz, Stephan P. A. Sauer

**Affiliations:** Department of Chemistry, University of Copenhagen, DK-2100 Copenhagen Ø, Denmark

In the original publication [1], there were mistakes in Table 3, Table 4, and Table 5 as published. The mistakes concern how the partition function of the hydroxyl radical OH was calculated from the data obtained with the wB97X-D/6-311G++(2df,2pd) calculations using the Gaussian16 program. This influenced all the rate constants calculated with transition state theory in these tables. The corrected Table 3, Table 4 and Table 5 appear below. The authors state that the scientific conclusions are unaffected. This correction was approved by the Academic Editor. The original publication has also been updated.

As a consequence of the errors in Table 3, Table 4, and Table 5, corrections have been made to the text of the following sections.

A correction has been made to the Abstract:

All the practically possible hydrogen abstraction reactions for guanosine and uridine have been investigated through quantum chemical calculations of energy barriers and rate constants. This was conducted at the level of density functional theory (DFT) with the ωB97X-D functional and the 6-311++G(2df,2pd) Pople basis set. Transition state theory with the Eckart tunneling correction was used to calculate the rate constants. The results show that the reaction involving the hydrogen labeled C4′ in the ribofuranose part has the largest rate constant for guanosine with the value 6.856 × 10^10^ L s^−1^mol^−1^ and the largest for uridine with the value 3.655 × 10^9^ L s^−1^mol^−1^. Based on the results for these two nucleosides, there is a noticeable similarity between the rate constants in the ribofuranose part of the molecule, even though they are bound to two entirely different nucleobases.

A correction has been made to 2. Results and Discussion, 2.3. Rate Constants, Paragraph 1:

In Table 3, we finally present the rate constants calculated for the abstraction of the different hydrogen atoms in guanosine and uridine. The results show that the reaction at C4’, i.e., in the RF part of the nucleoside, has the largest rate constant for both molecules at 6.856 × 10^10^ L s^−1^mol^−1^ and 3.655 × 10^9^ L s^−1^mol^−1^ for guanosine and uridine, respectively, followed by the abstraction of a hydrogen from C2’—again in the RF part. When looking at the other calculated properties, one observes that these reactions also have the lowest ΔG^‡^ values for both molecules. This inverse relationship between the rate constant and ΔG^‡^ can be noticed throughout all the reactions, showing that the energy term in the TST equation for the rate constant, Equation (1), is the dominant term.

A correction has been made to 2. Results and Discussion, 2.3. Rate Constants, Paragraph 5:

We can also compare, in Table 4 and Table 5, our rate constants for the hydrogen abstraction reactions in the nucleobase part of the nucleosides to the previous results for the hydrogen abstraction reactions in the nucleobases themselves [9]. In general, the changes in the rate constants due to the RF part are relatively small. With the exception of the abstraction of H8 in guanosine/guanine, they differ by less than one order of magnitude. For uridine/uracil, the rate constants are larger in the isolated uracil molecule compared to the rate constants for the same hydrogens. Meanwhile, for guanosine/guanine, the opposite is true for all but the H8 hydrogen abstraction reaction.

A correction has been made to 4. Conclusions, Paragraph 1:

We have systematically investigated all the hydrogen abstraction reactions in the two nucleosides, guanosine and uridine, with DFT calculations at the ωB97X-D/6-311G++(2df,2pd) level. The investigation has shown that, in general, the most favorable hydrogen abstraction reactions occur on the carbon-bound hydrogens in the ribofuranose part of the nucleosides and not in the nucleobase part. The fastest hydrogen abstraction reaction is the one that occurs on the C4’ hydrogen, with a rate constant of 6.856 × 10^10^ L s^−1^mol^−1^ for guanosine and 3.655 × 10^9^ L s^−1^mol^−1^ for uridine. These two reactions also have the lowest ΔG^‡^, indicating that the rate constants are dominated by the exponential energy term.

The authors state that the scientific conclusions are unaffected. This correction was approved by the Academic Editor. The original publication has also been updated.

## Figures and Tables

**Table 3:** Rate constants for the different hydrogens in guanosine and uridine (L s^−1^mol^−1^) calculated at the ωB97X-D/6-311G++(2df,2pd) level.

Hydrogen	Guanosine	Hydrogen	Uridine
C1′	1.603 × 10^9^	C1′	7.037 × 10^8^
C2′	1.225 × 10^10^	C2′	1.326 × 10^9^
C3′	6.667 × 10^8^	C3′	2.942 × 10^8^
C4′	6.856 × 10^10^	C4′	3.655 × 10^9^
C5′_HC	7.123 × 10^8^	C5′_HC	4.380 × 10^8^
C5′_HO	1.830 × 10^9^	C5′_HO	5.316 × 10^8^
O2	9.078 × 10^8^	O2	4.386 × 10^5^
O3	2.543 × 10^9^	O3	2.096 × 10^5^
H1	5.034 × 10^9^	H3	1.527 × 10^2^
H8	1.048 × 10^7^	H5	1.878 × 10^5^
H21	7.202 × 10^9^	H6	5.836 × 10^4^
H22	2.795 × 10^9^	N/A	N/A

**Table 4:** Comparison of rate constants in guanosine and guanine (L s^−1^mol^−1^).

Hydrogen	Guanosine	Guanine
H1	5.034 × 10^9^	6.925 × 10^8^
H8	1.048 × 10^7^	8.732 × 10^5^
H21	7.202 × 10^9^	1.036 × 10^10^
H22	2.795 × 10^9^	7.347 × 10^8^

**Table 5:** Comparison of rate constants in uridine and uracil (L s^−1^mol^−1^).

Hydrogen	Uridine	Uracil
H3	1.527 × 10^2^	2.430 × 10^2^
H5	1.878 × 10^5^	2.987 × 10^5^
H6	5.836 × 10^4^	9.281 × 10^4^

## References

[1] Schaltz K.F., Sauer S.P.A. (2023). A Theoretical Study of Hydrogen Abstraction Reactions in Guanosine and Uridine. Int. J. Mol. Sci..

