# Supplementary material for: A Theoretical Study of Hydrogen Abstraction Reactions in Guanosine and Uridine
Source: Int J Mol Sci. 2023 May 3;24(9):8192. doi: 10.3390/ijms24098192 (PMC10179689; doi:10.3390/ijms24098192)
Supplement: Supplementary file 1 [file ijms-24-08192-s001.zip › ijms-2324838-supplementary.pdf]

Supporting information

to

A theoretical study of hydrogen abstraction reactions in guanosine and uridine

by

Kasper Frølund Schaltz and Stephan P. A. Sauer

Department of Chemistry, University of Copenhagen, DK-2100 Copenhagen Ø, Denmark

Intrinsic Reaction Coordinate plots – Uridine

C1

Forward and reverse:

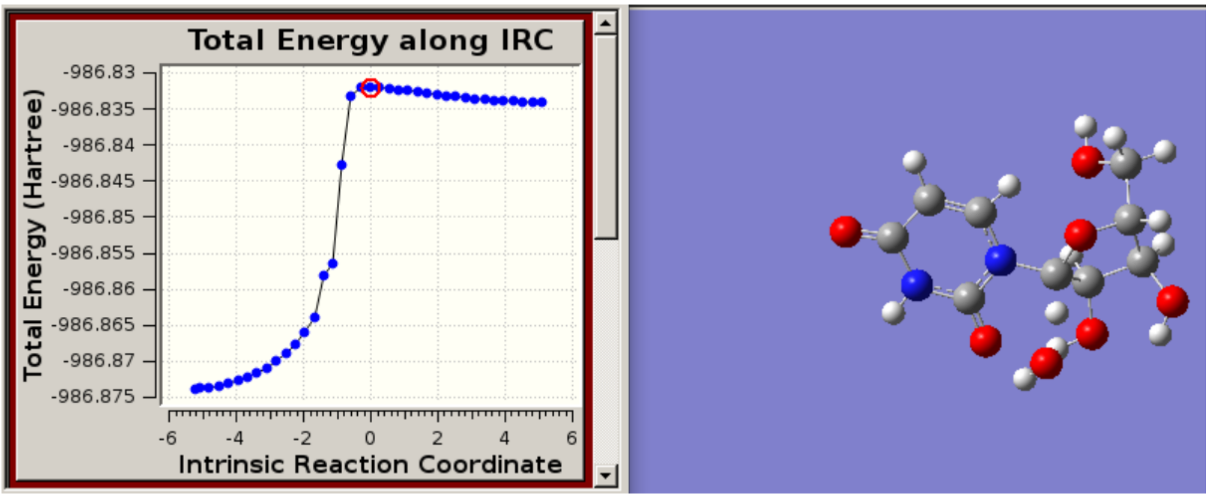

C2

Reverse:

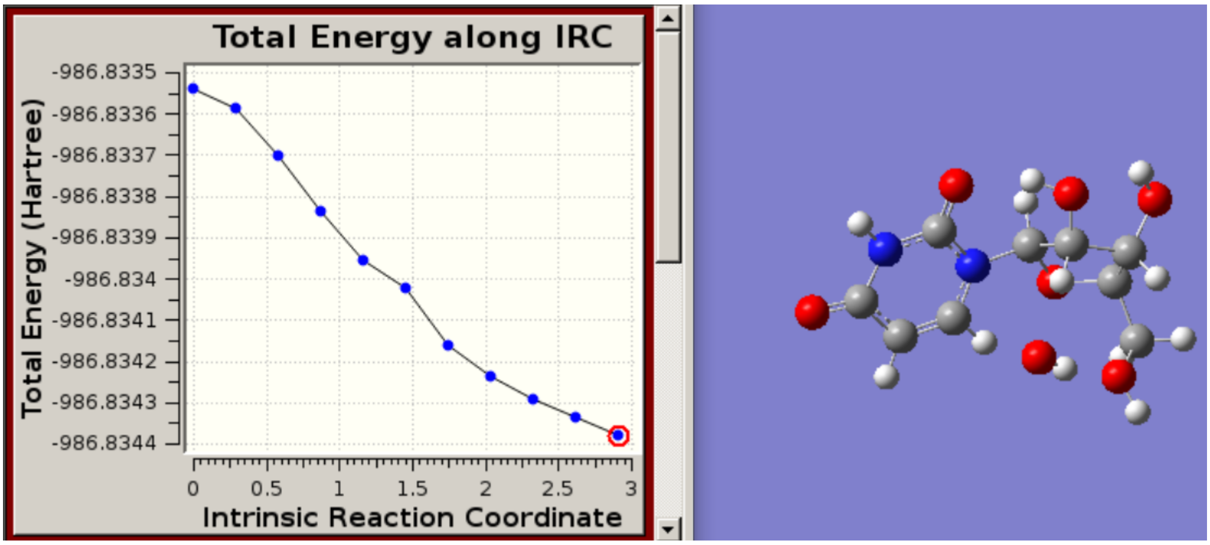

Forward:

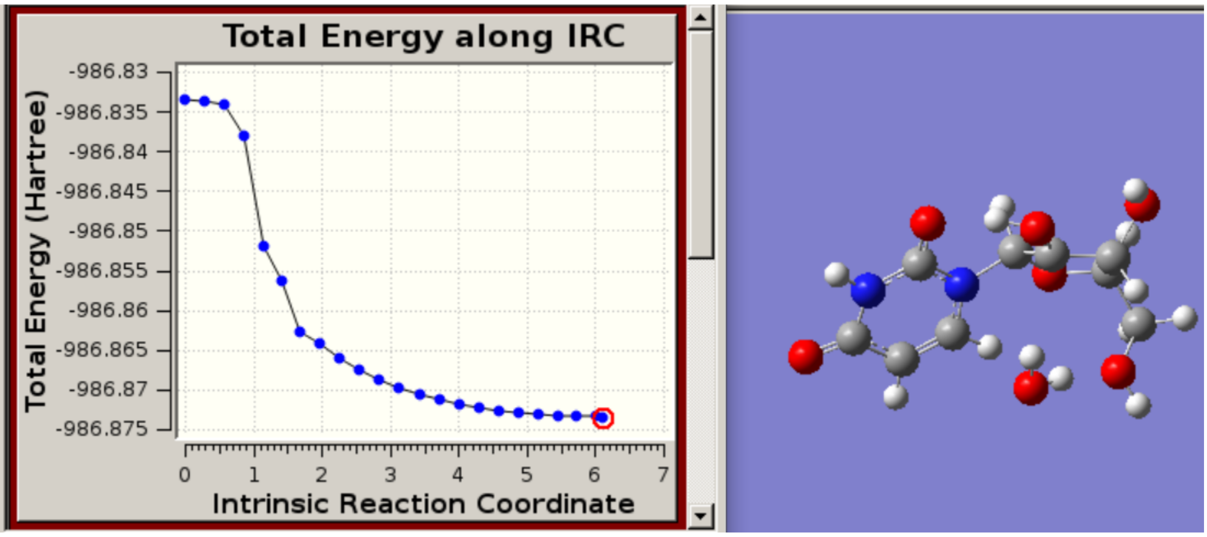

C3

Reverse:

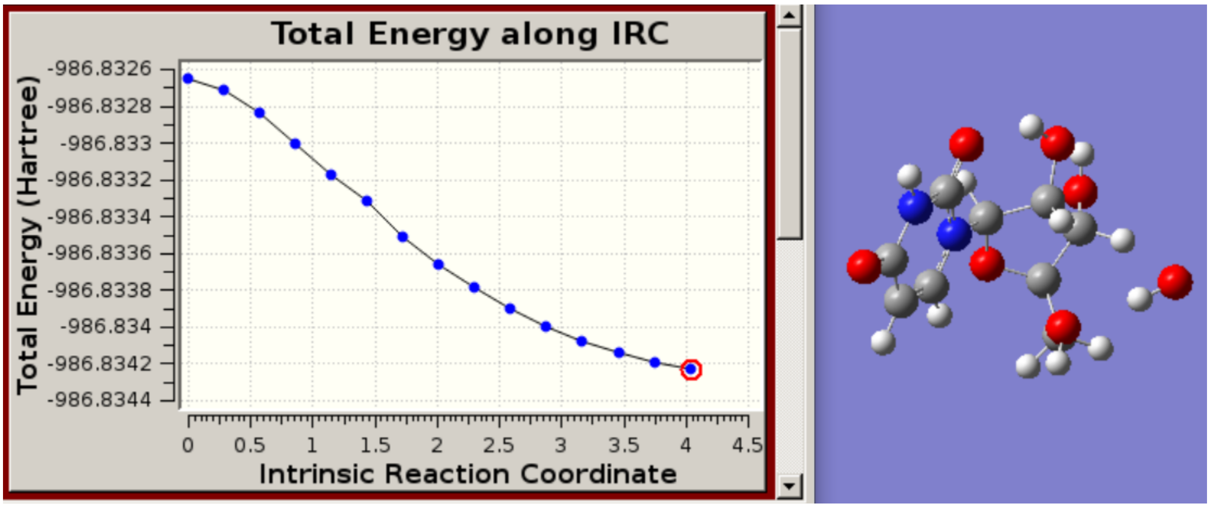

Forward:

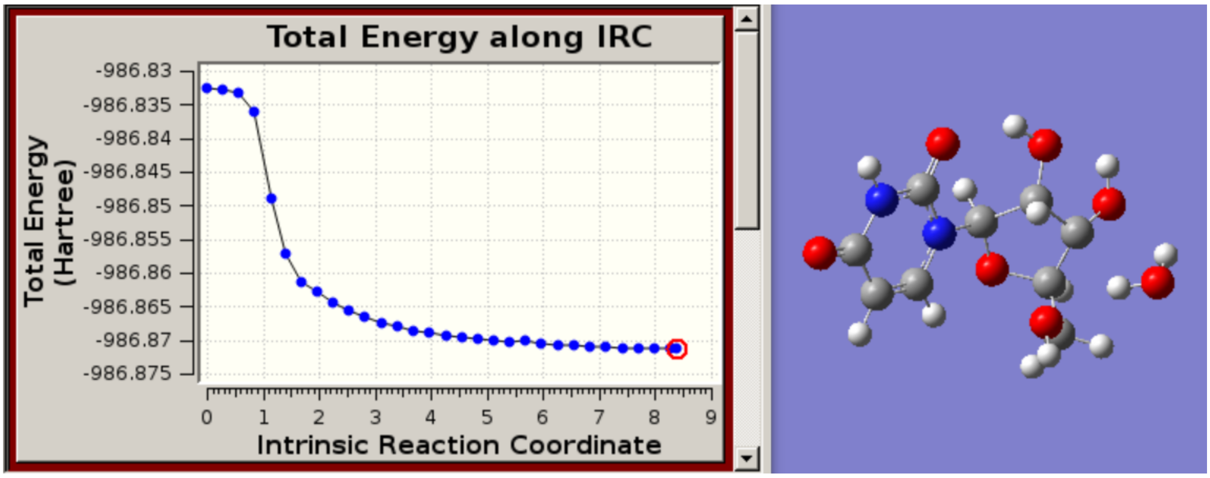

C4

Reverse and forward:

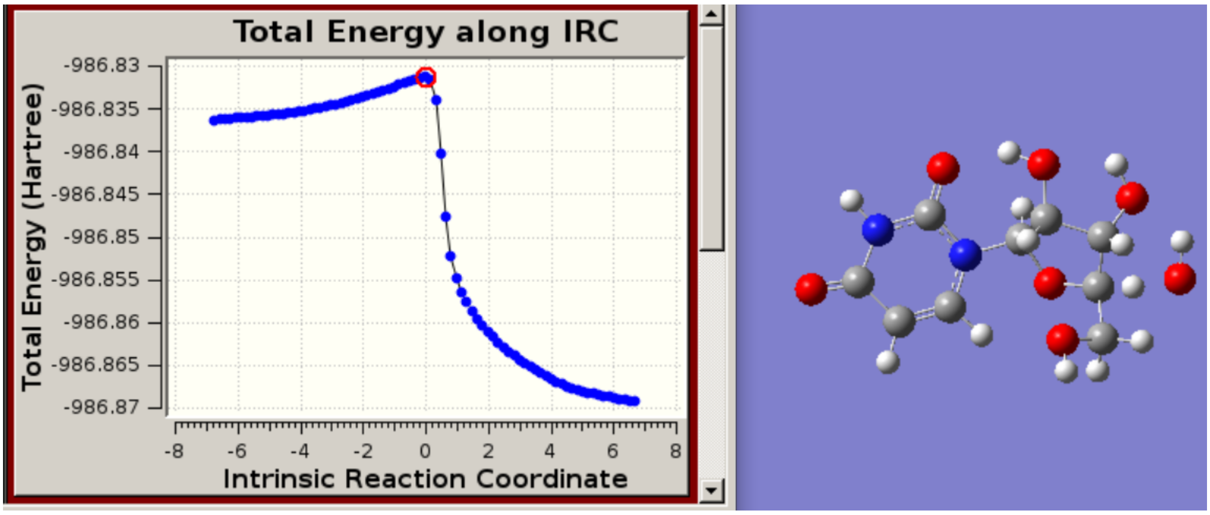

C5_HO

Forward and reverse:

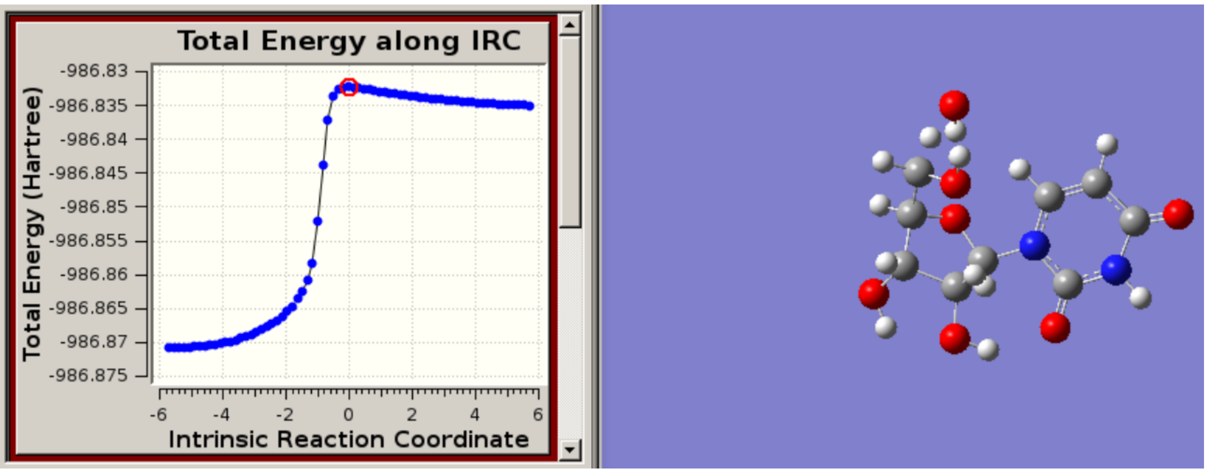

C5_HC

Forward and reverse

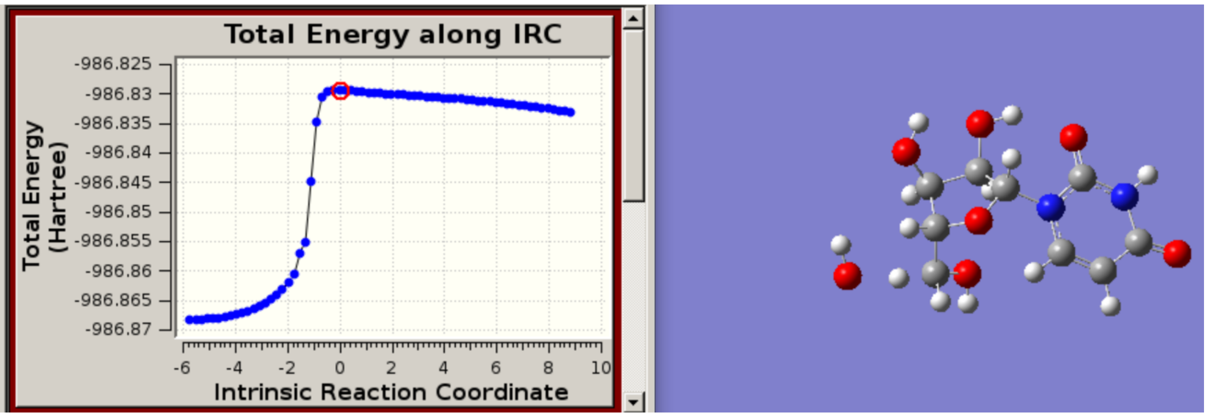

O2

Forward and reverse:

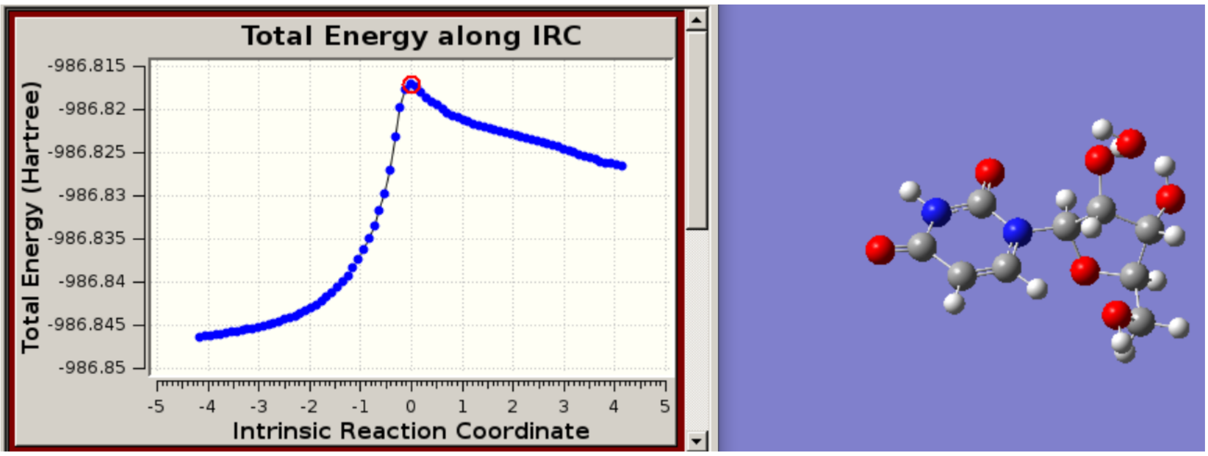

O3

Reverse and forward:

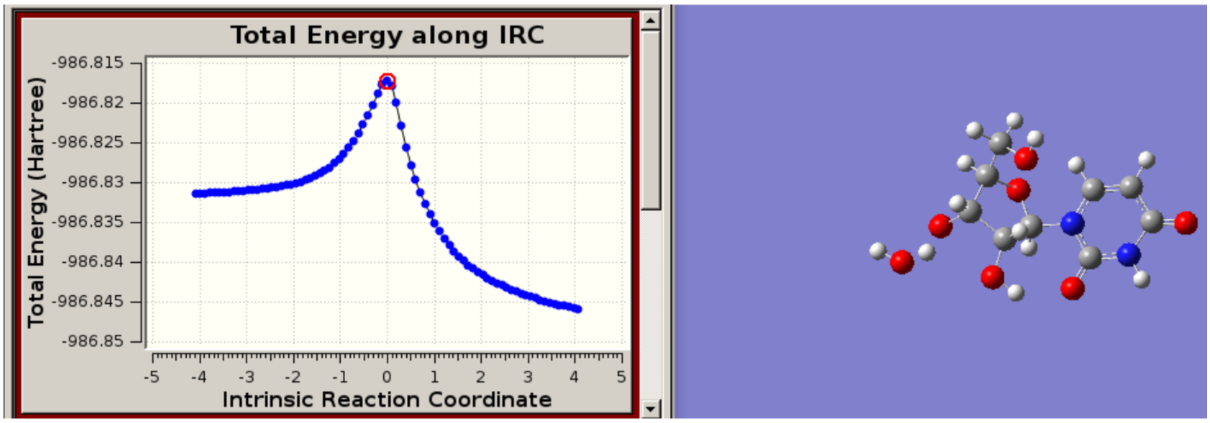

H3

Reverse:

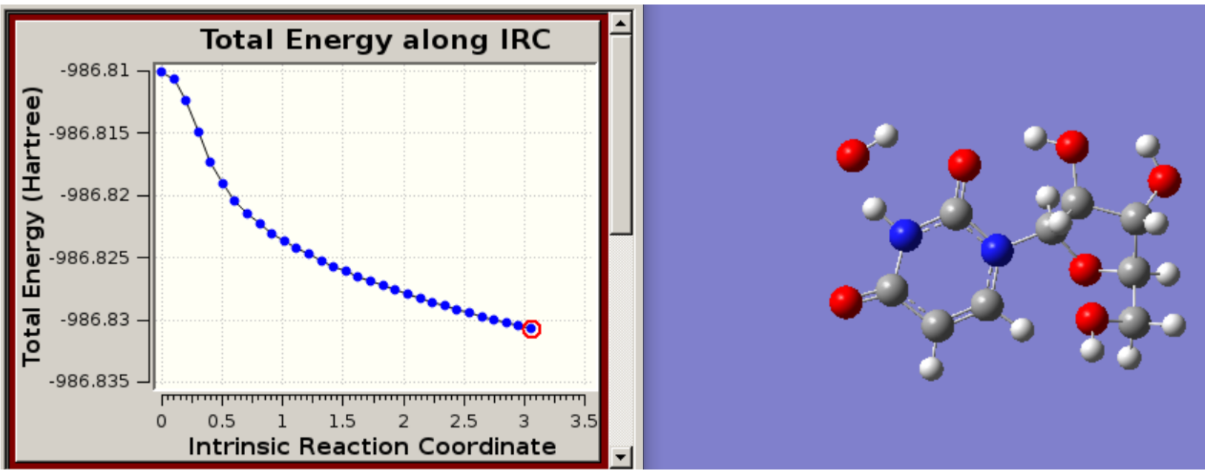

Forward:

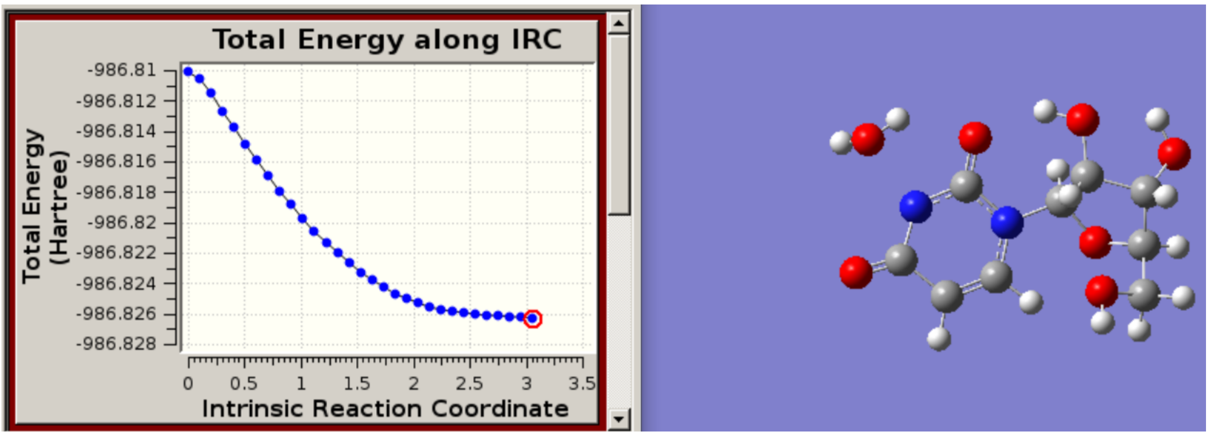

H5

Reverse:

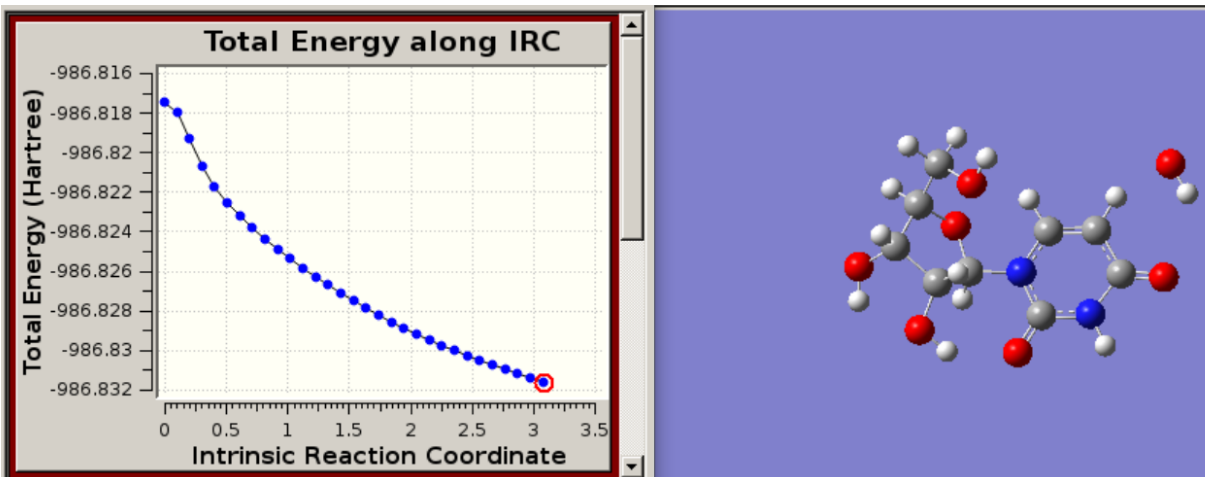

Forward:

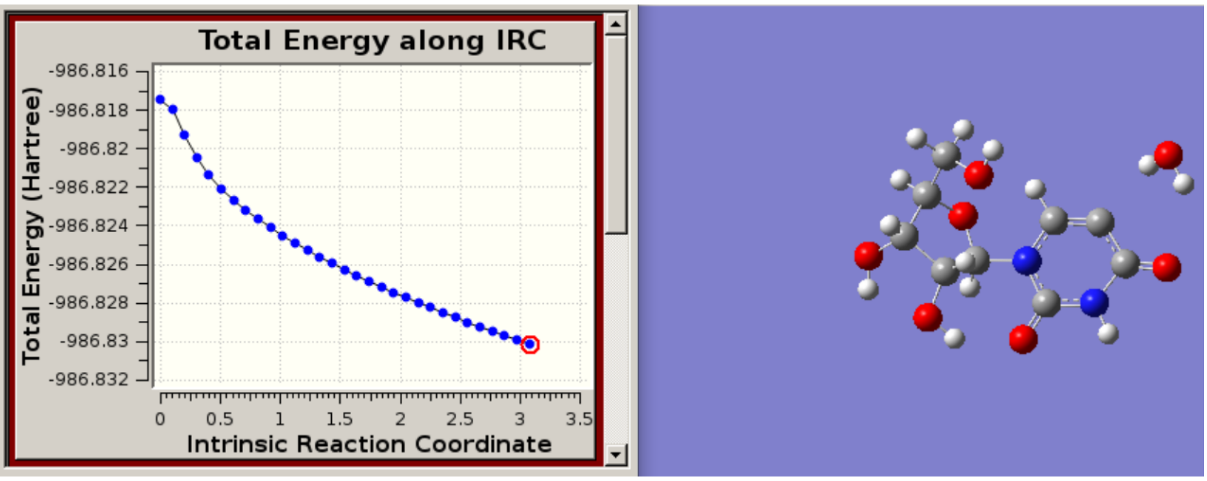

H6

Reverse:

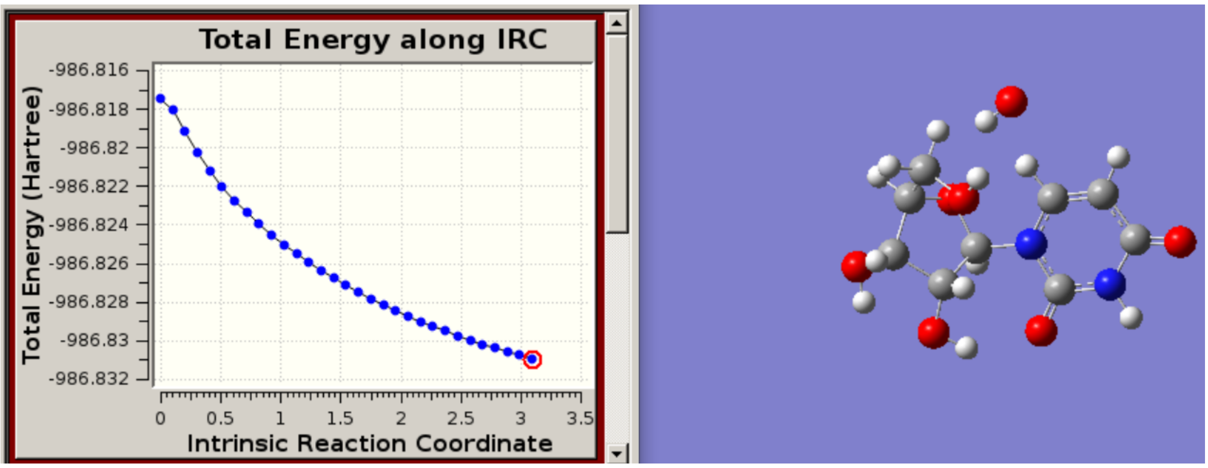
Total Energy along IRC
Total Energy (Hartree)
-986.816
-986.818
-986.82
-986.822
-986.824
-986.826
-986.828
-986.83
-986.832
0
0.5
1
1.5
2
2.5
3
3.5
Intrinsic Reaction Coordinate

Forward:

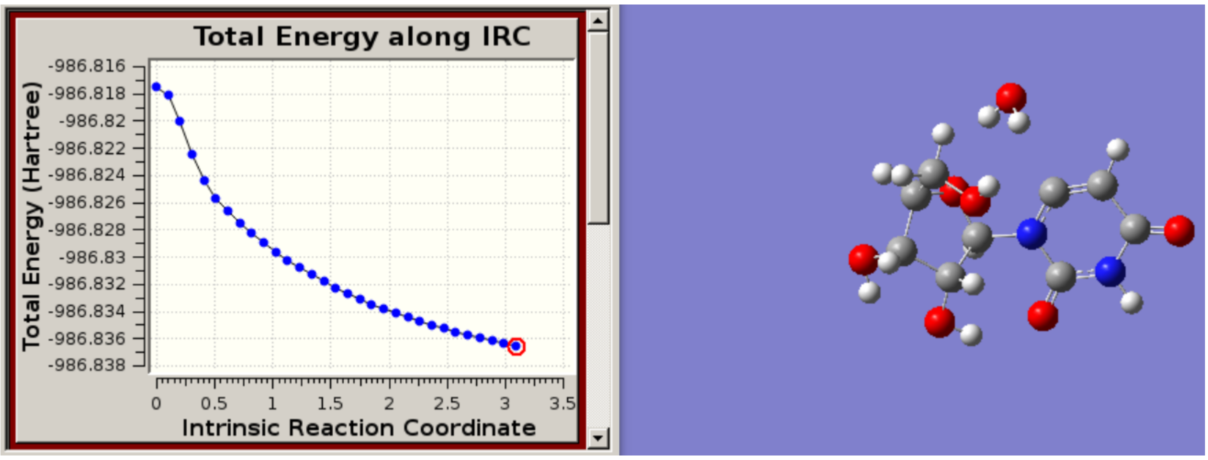
Total Energy along IRC
Total Energy (Hartree)
-986.816
-986.818
-986.82
-986.822
-986.824
-986.826
-986.828
-986.83
-986.832
-986.834
-986.836
-986.838
0
0.5
1
1.5
2
2.5
3
3.5
Intrinsic Reaction Coordinate

Intrinsic Reaction Coordinate plots – Guanosine

C1

Reverse:

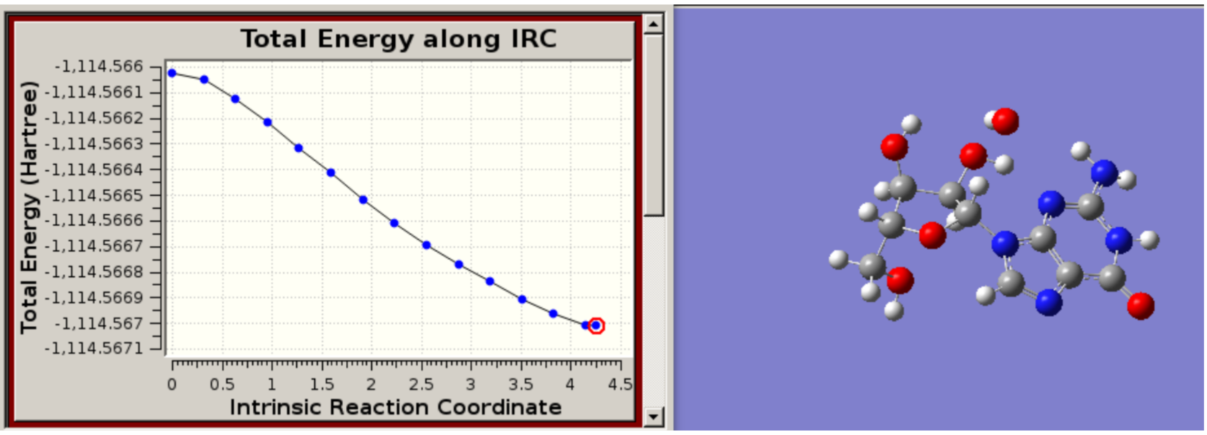

Forward:

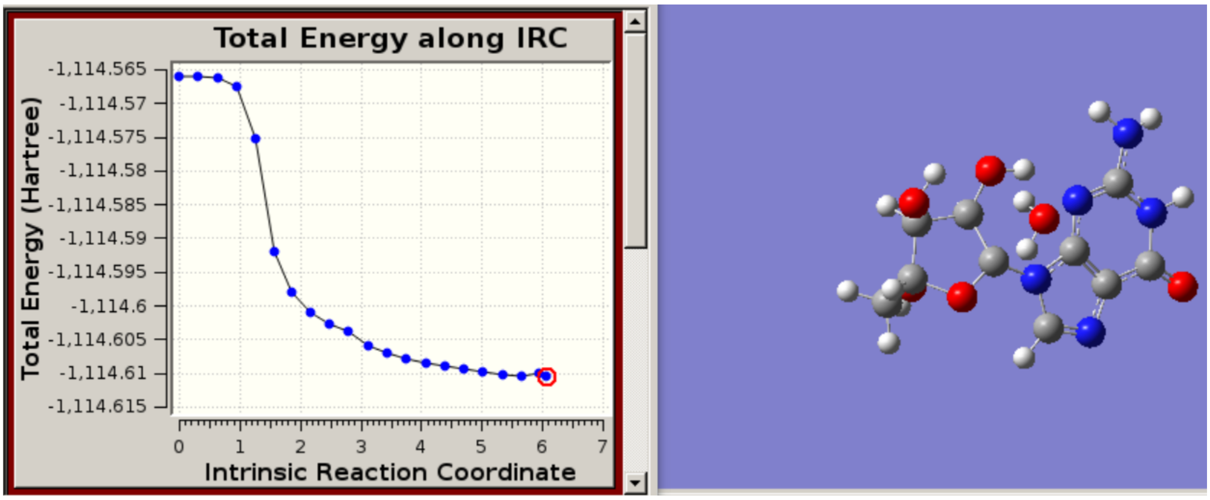

C2

Reverse:

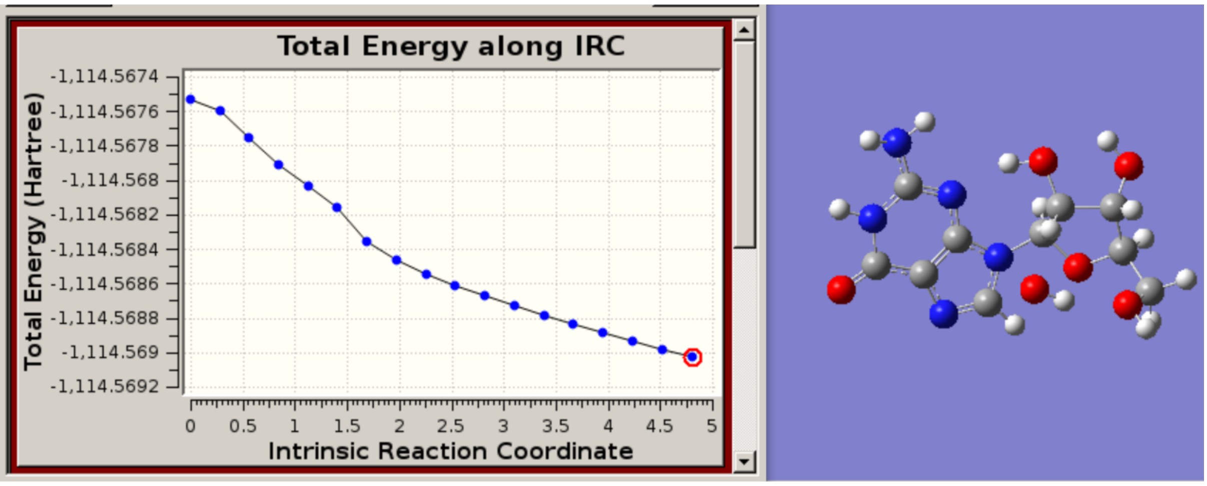

Forward:

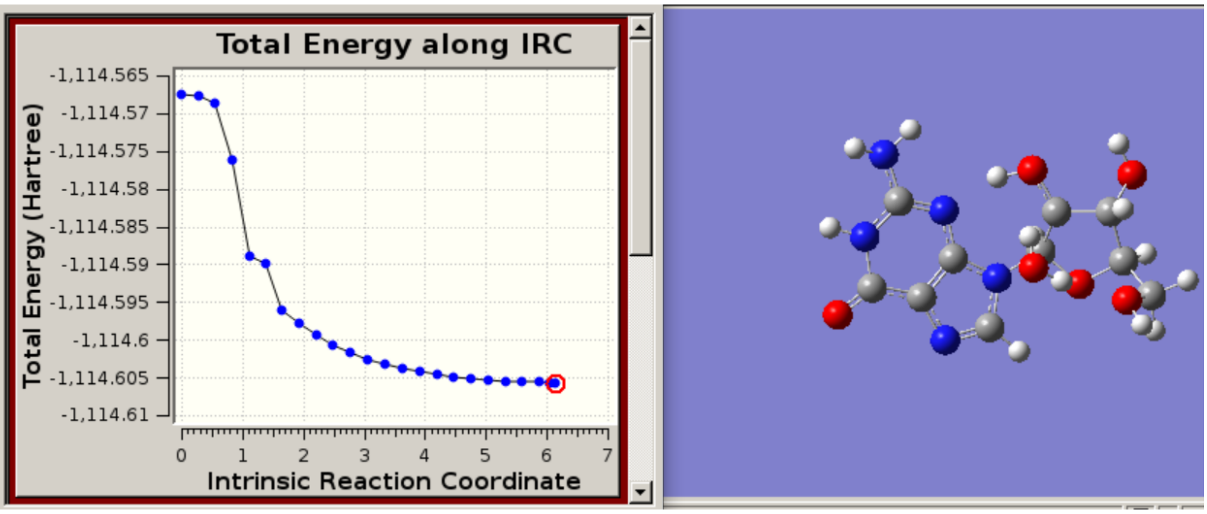

C3

Reverse:

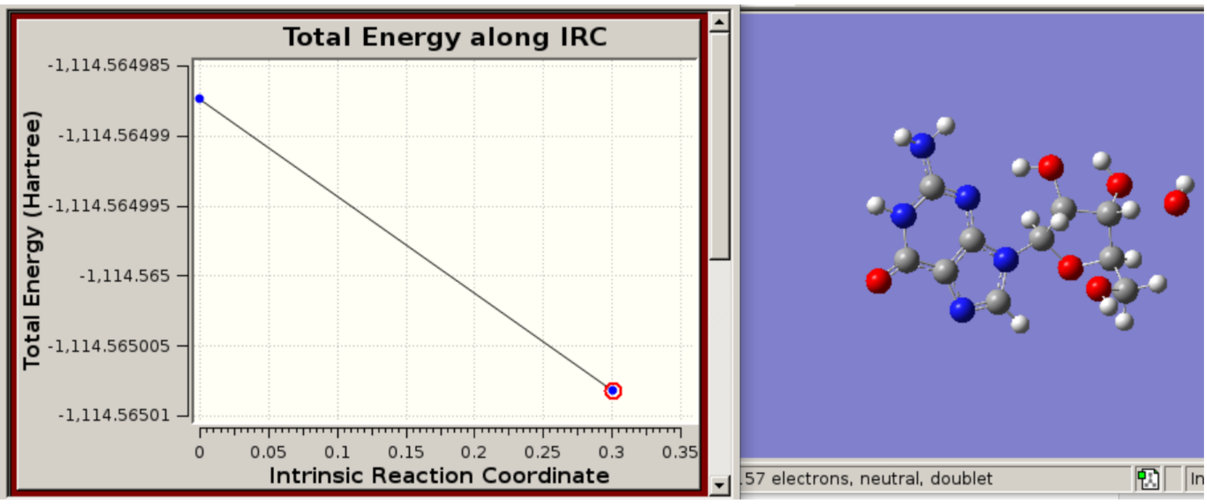

Forward:

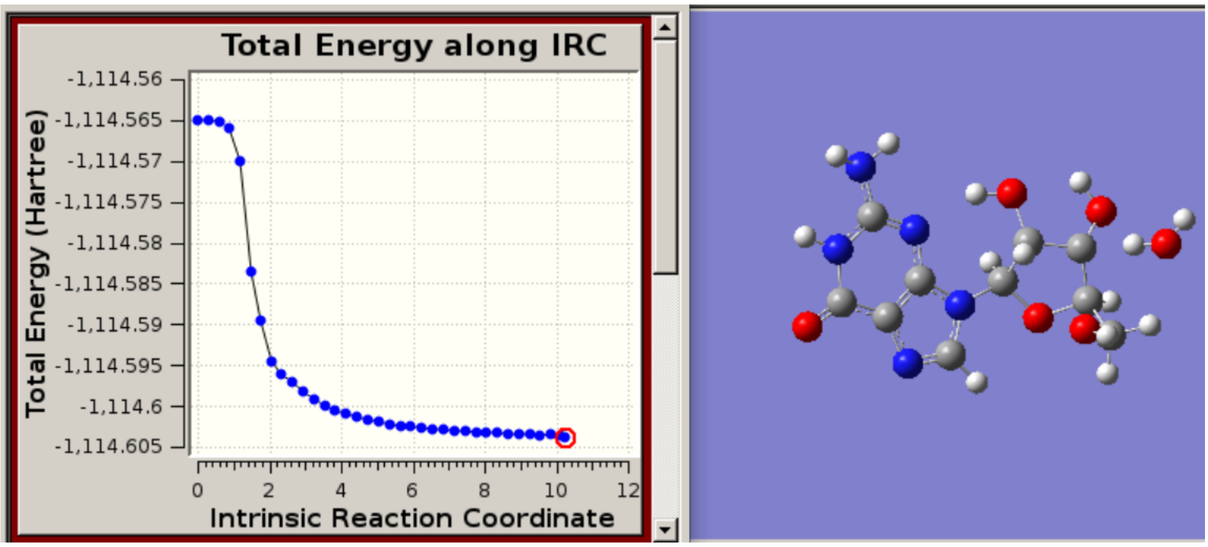

C4

Reverse:

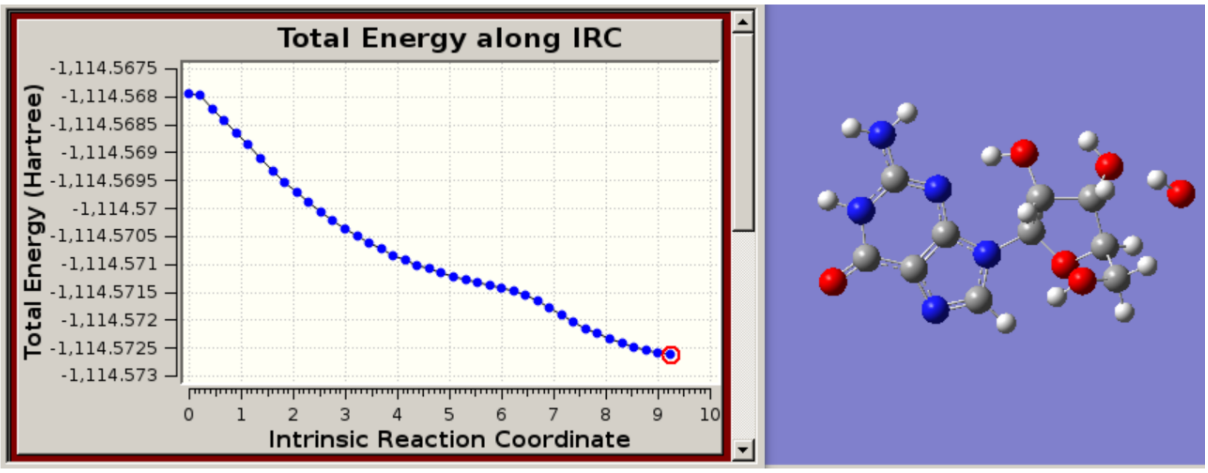

Forward:

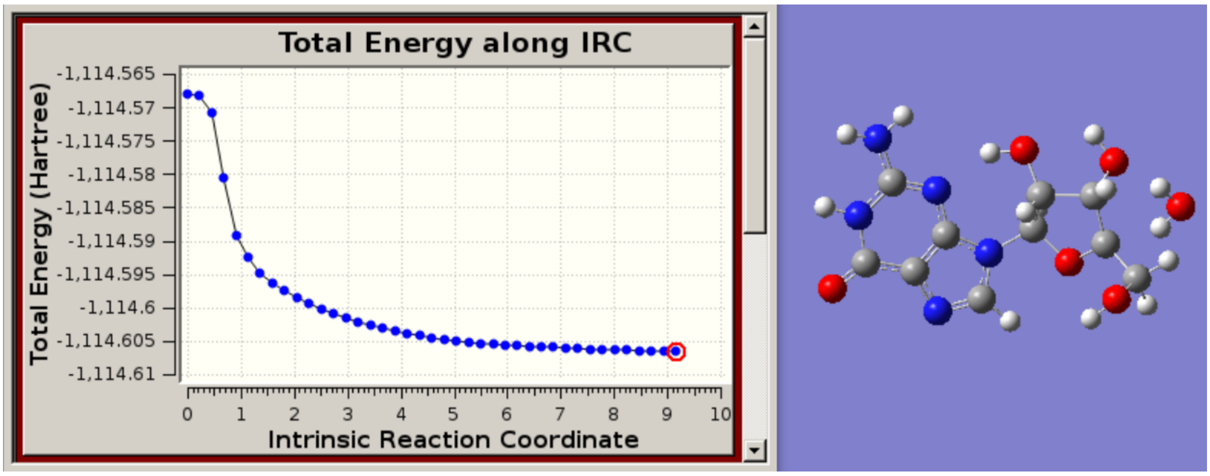

C5_HC

Reverse:

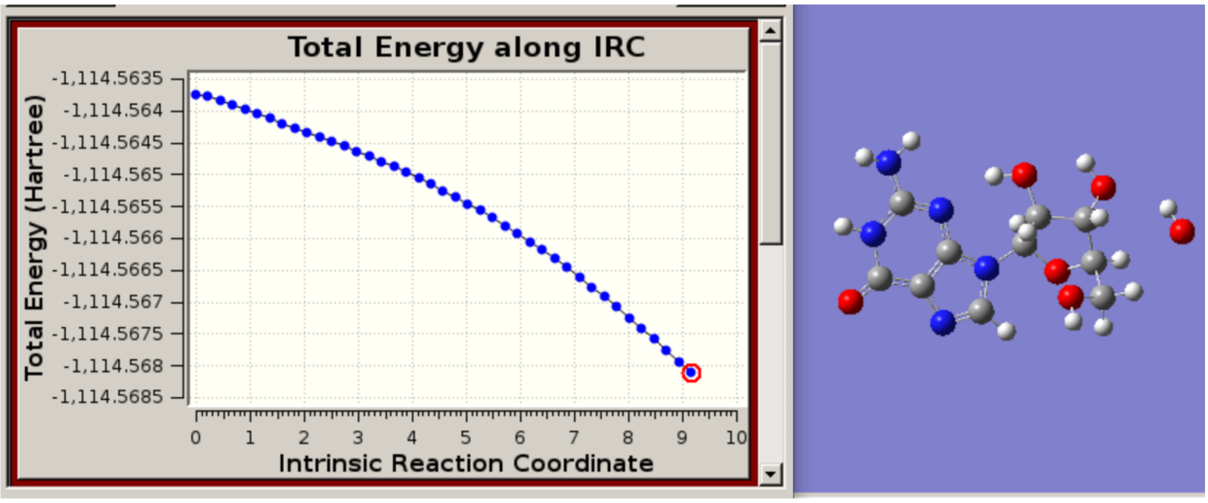

Forward:

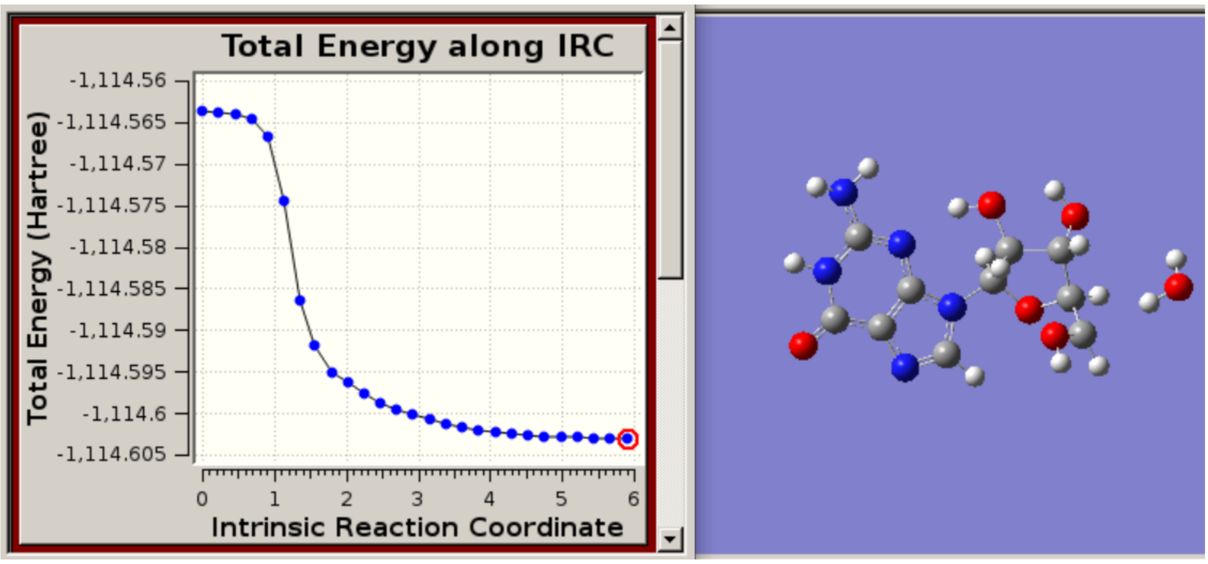

C5_HO

Reverse:

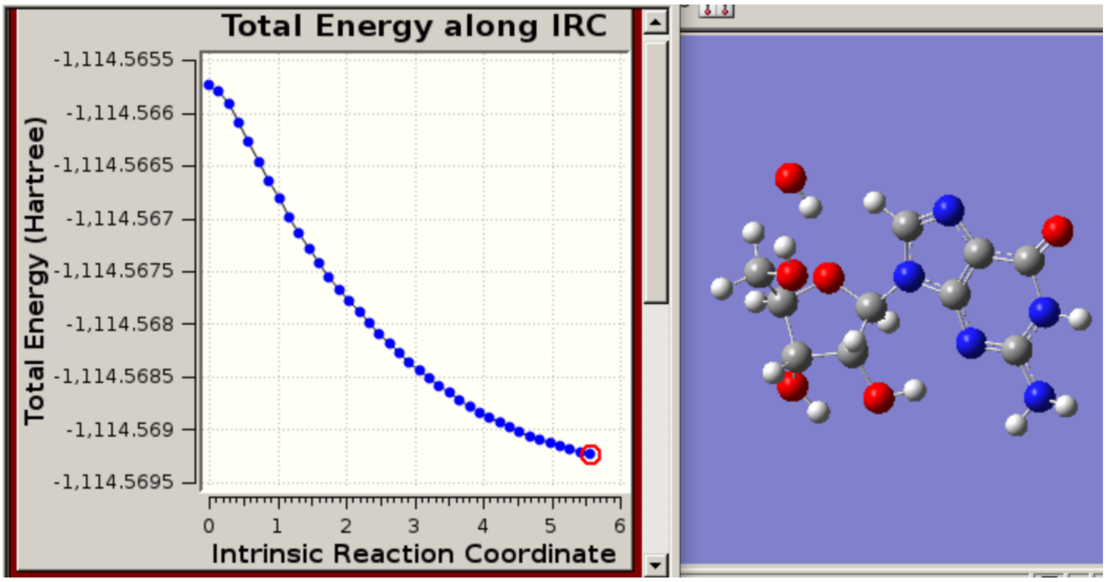

Forward:

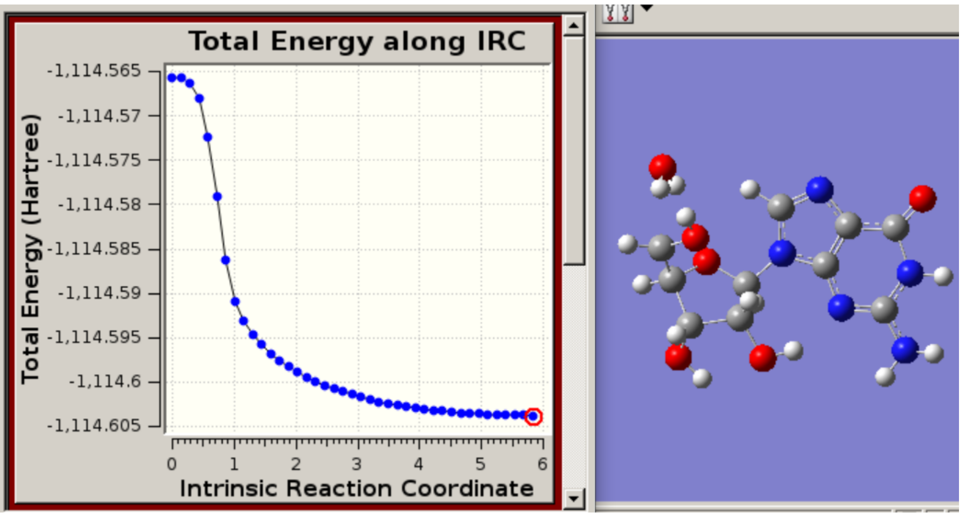

O2

Reverse:

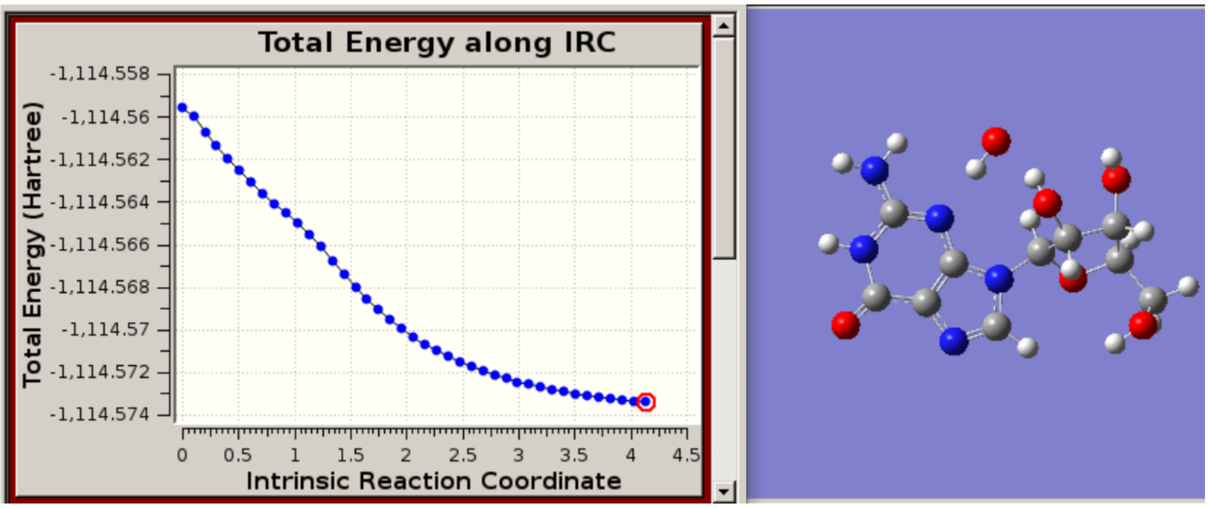

Forward:

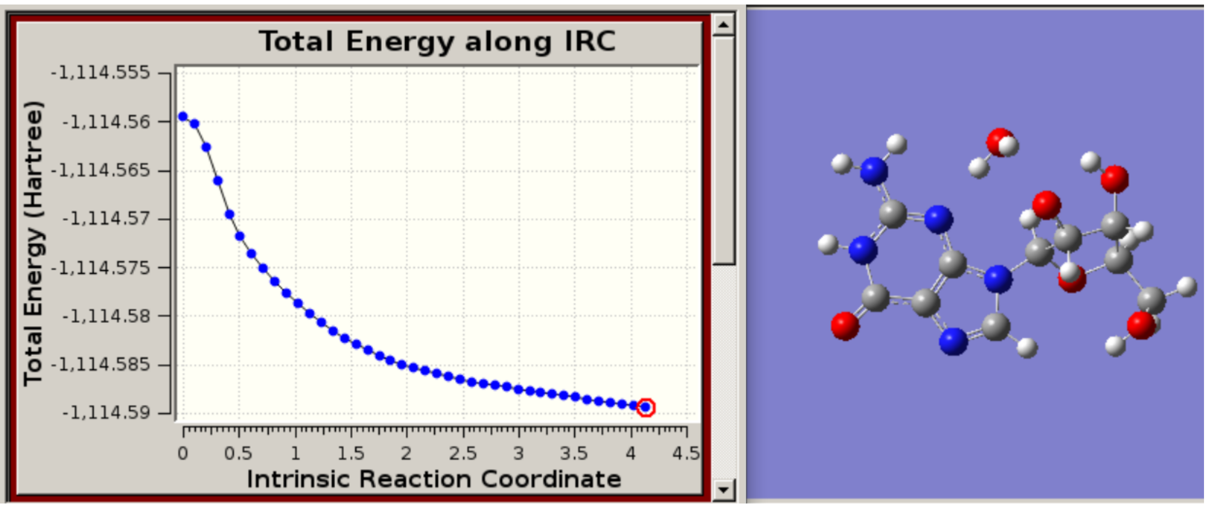

O3

Reverse:

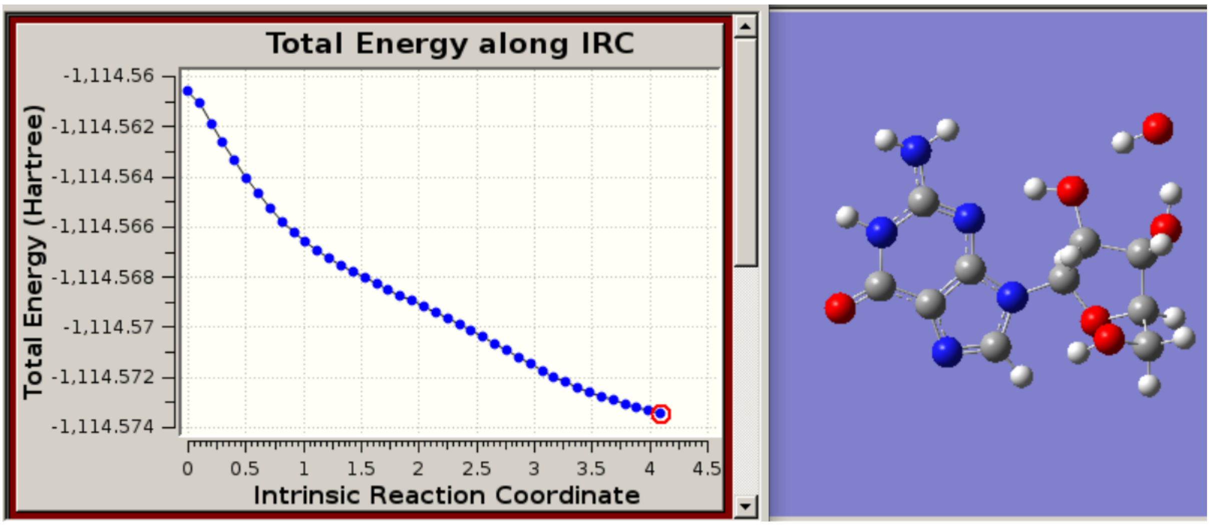

Forward:

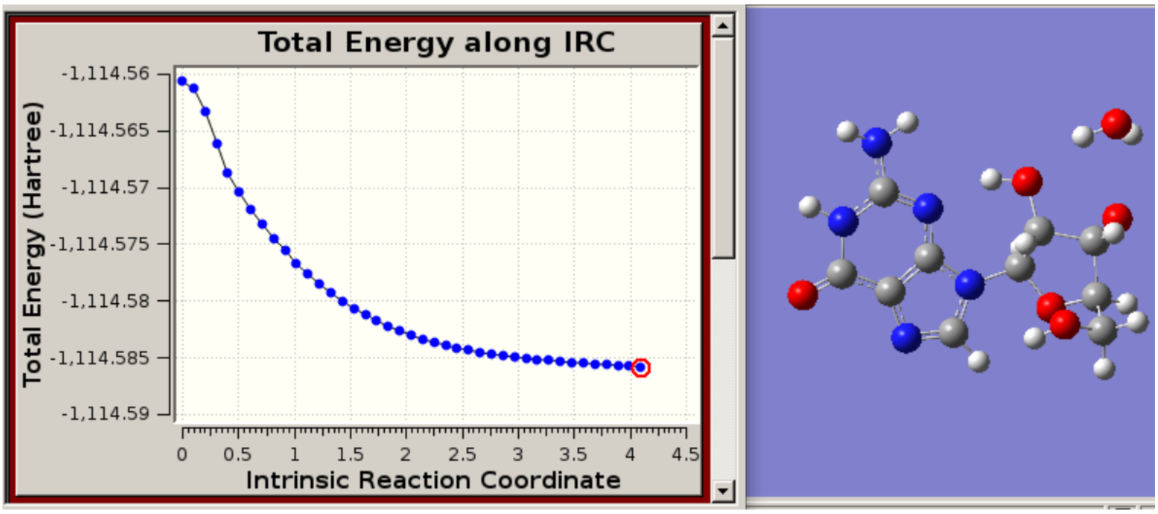

H1

Reverse:

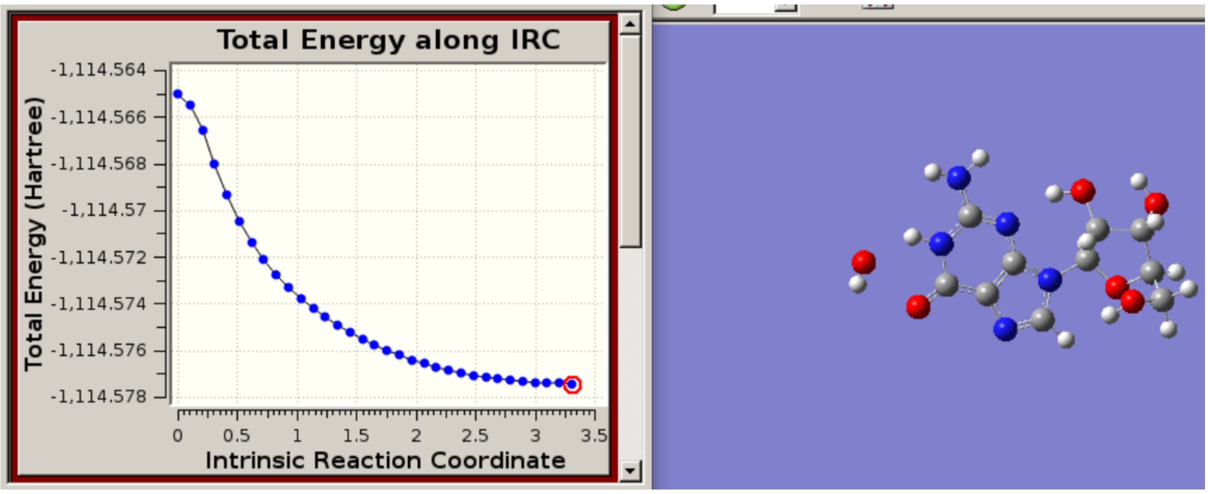

Forward:

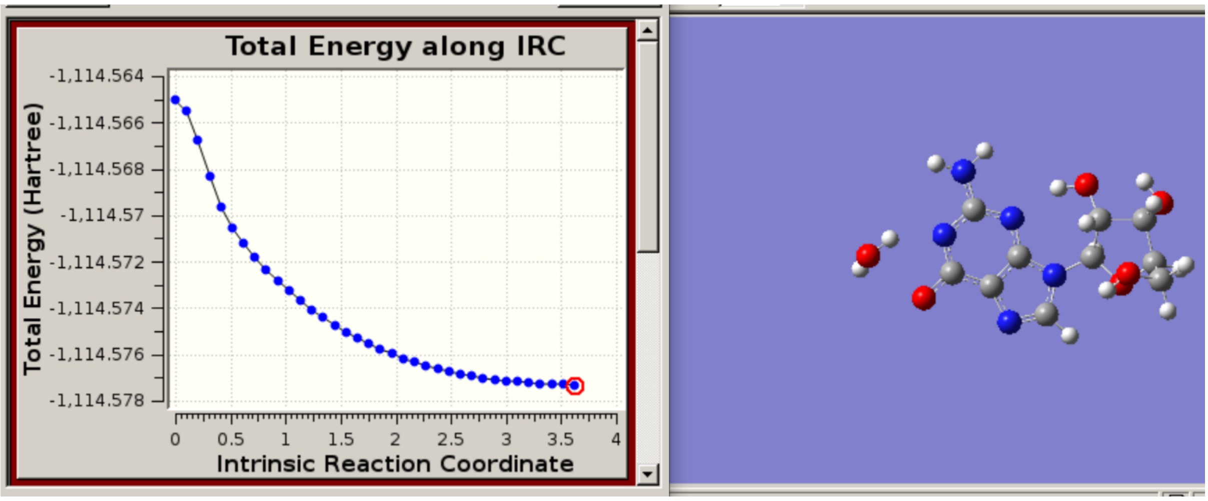

H8

Reverse:

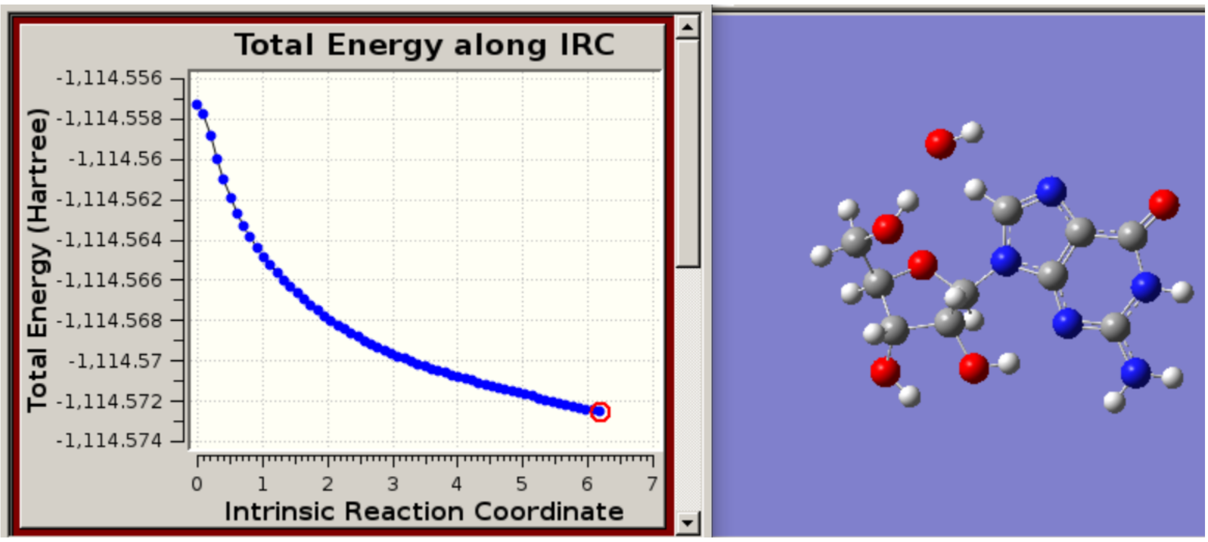

Forward:

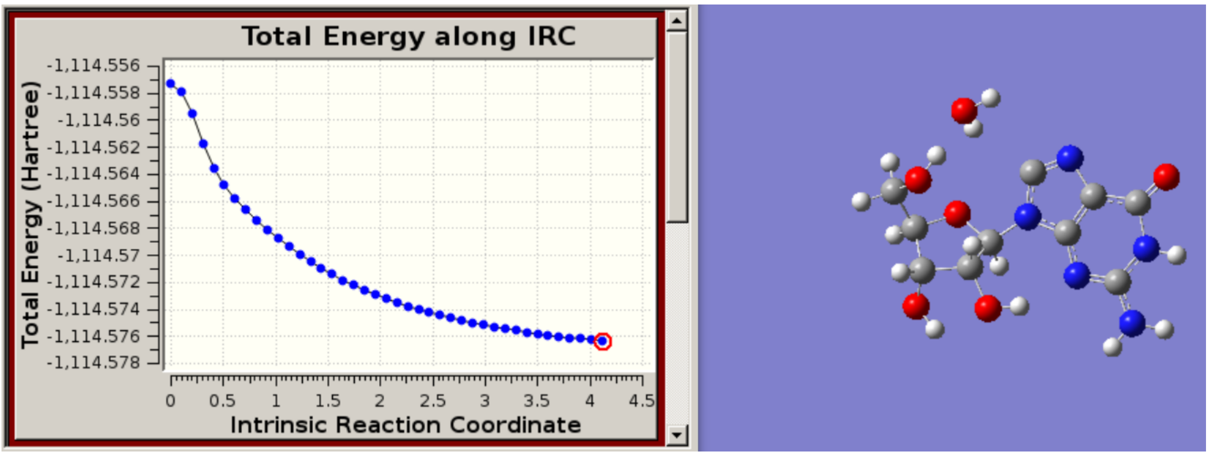

H21

Reverse:

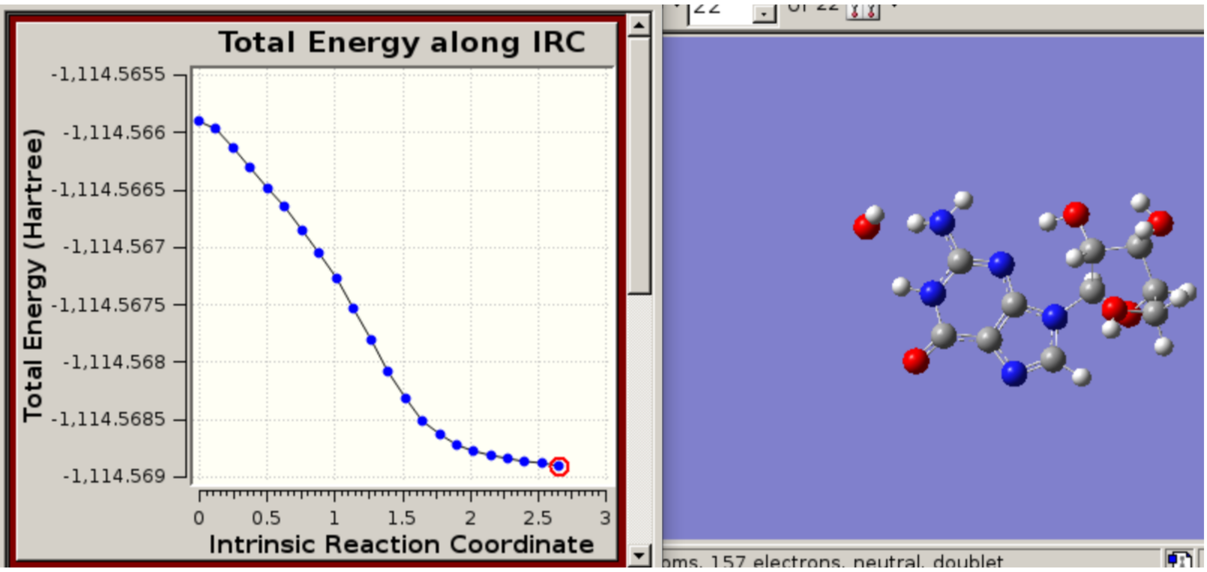

Forward:

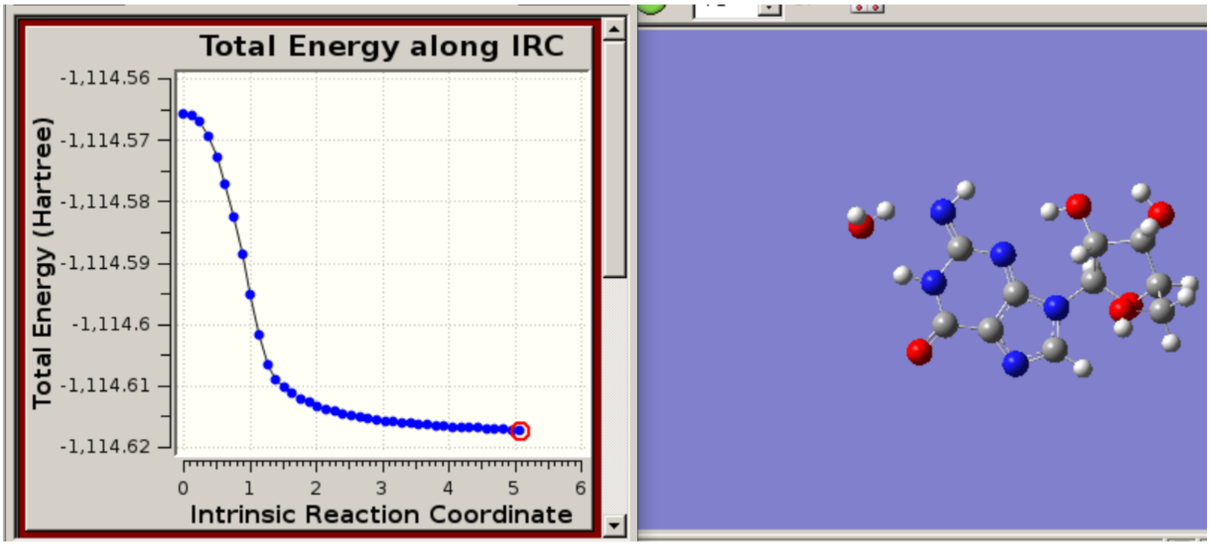

H22

Reverse:

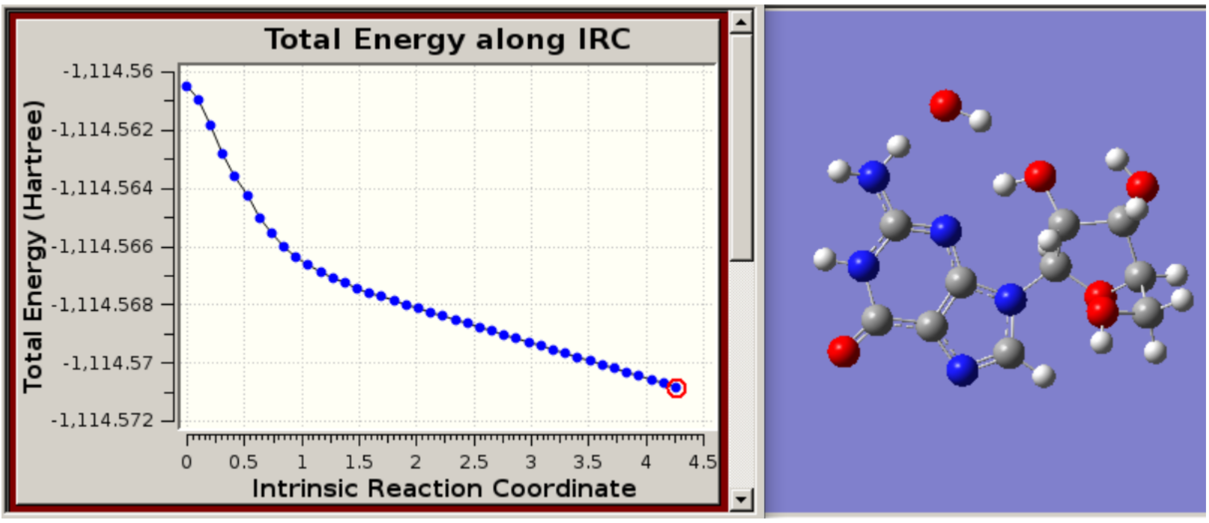
Total Energy along IRC
Total Energy (Hartree)
-1,114.56
-1,114.562
-1,114.564
-1,114.566
-1,114.568
-1,114.57
-1,114.572
0
0.5
1
1.5
2
2.5
3
3.5
4
4.5
Intrinsic Reaction Coordinate

Forward:

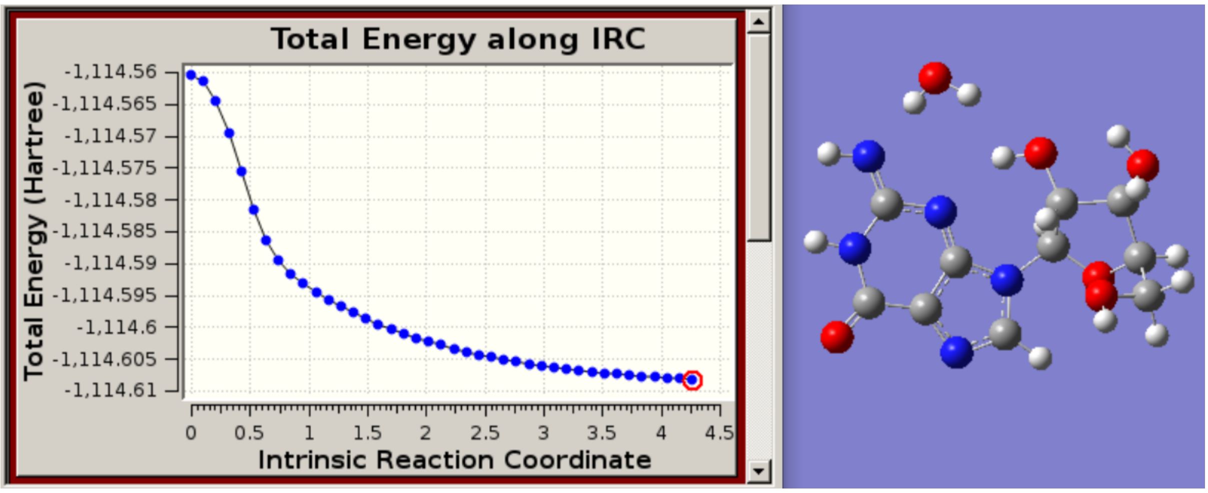
Total Energy along IRC
Total Energy (Hartree)
-1,114.56
-1,114.565
-1,114.57
-1,114.575
-1,114.58
-1,114.585
-1,114.59
-1,114.595
-1,114.6
-1,114.605
-1,114.61
0
0.5
1
1.5
2
2.5
3
3.5
4
4.5
Intrinsic Reaction Coordinate
